# Supplementary material for: Alcohol consumption and associated risk factors in Burkina Faso: results of a population-based cross-sectional survey
Source: BMJ Open. 2022 Feb 10;12(2):e058005. doi: 10.1136/bmjopen-2021-058005 (PMC8845319; doi:10.1136/bmjopen-2021-058005)
Supplement: Author's manuscript [file bmjopen-2021-058005.draft_revisions.pdf]

BMJ Open

BMJ Open is committed to open peer review. As part of this commitment we make the peer review history of every article we publish publicly available.

When an article is published we post the peer reviewers' comments and the authors' responses online. We also post the versions of the paper that were used during peer review. These are the versions that the peer review comments apply to.

The versions of the paper that follow are the versions that were submitted during the peer review process. They are not the versions of record or the final published versions. They should not be cited or distributed as the published version of this manuscript.

BMJ Open is an open access journal and the full, final, typeset and author-corrected version of record of the manuscript is available on our site with no access controls, subscription charges or pay-per-view fees (http://bmjopen.bmj.com).

If you have any questions on BMJ Open's open peer review process please email info.bmjopen@bmj.com

BMJ Open

Alcohol consumption and associated risk factors in Burkina Faso: results of a population-based cross-sectional survey.

Journal:	*BMJ Open*
Manuscript ID	bmjopen-2021-058005
Article Type:	Original research
Date Submitted by the Author:	05-Oct-2021
Complete List of Authors:	Bonnechère, Bruno; Hasselt University, REVAL Rehabilitation Research Center<br>Samadoulougou, Sékou; Quebec Heart and Lung Institute Research Center; Laval University<br>Cisse, Kadari; Institut de Recherche en Sciences de la Sante, Biomedical et santé publique; Université Libre de Bruxelles- Ecole de santé publique, Centre de Recherche en Epidémiologie, Biostatistiques et Recherche Clinique<br>Tassembedo, Souleymane; Centre MURAZ, Programme de Recherche sur les maladies Infectieuses<br>Kouanda, Seni; Institut de Recherche en Sciences de la Sante<br>Kirakoya-Samadoulougou, Fati; Université Libre de Bruxelles, Biostatistics, and Clinical Research
Keywords:	EPIDEMIOLOGY, PUBLIC HEALTH, TOXICOLOGY

SCHOLARONE™
Manuscripts

I, the Submitting Author has the right to grant and does grant on behalf of all authors of the Work (as defined in the below author licence), an exclusive licence and/or a non-exclusive licence for contributions from authors who are: i) UK Crown employees; ii) where BMJ has agreed a CC-BY licence shall apply, and/or iii) in accordance with the terms applicable for US Federal Government officers or employees acting as part of their official duties; on a worldwide, perpetual, irrevocable, royalty-free basis to BMJ Publishing Group Ltd ("BMJ") its licensees and where the relevant Journal is co-owned by BMJ to the co-owners of the Journal, to publish the Work in this journal and any other BMJ products and to exploit all rights, as set out in our licence.

The Submitting Author accepts and understands that any supply made under these terms is made by BMJ to the Submitting Author unless you are acting as an employee on behalf of your employer or a postgraduate student of an affiliated institution which is paying any applicable article publishing charge ("APC") for Open Access articles. Where the Submitting Author wishes to make the Work available on an Open Access basis (and intends to pay the relevant APC), the terms of reuse of such Open Access shall be governed by a Creative Commons licence – details of these licences and which Creative Commons licence will apply to this Work are set out in our licence referred to above.

Other than as permitted in any relevant BMJ Author's Self Archiving Policies, I confirm this Work has not been accepted for publication elsewhere, is not being considered for publication elsewhere and does not duplicate material already published. I confirm all authors consent to publication of this Work and authorise the granting of this licence.

For peer review only

Alcohol consumption and associated risk factors in Burkina Faso: results of a population-based cross-sectional survey.

Bonnechère Bruno[1*], Samadoulougou Sékou[2,3], Cissé Kadari[4,5],Tassembedo Souleymane[6], Kouanda Seni[5,7], Kirakoya-Samadoulougou Fati[4]

[1] REVAL Rehabilitation Research Center, Faculty of Rehabilitation Sciences, Hasselt University, Diepenbeek, Belgium

[2] Evaluation Platform on Obesity Prevention, Quebec Heart and Lung Institute Research Center, Quebec City, QC G1V 4G5, Canada

[3] Centre for Research on Planning and Development (CRAD), Laval University, Quebec, G1V 0A6, Canada

[4] Centre de Recherche en Epidémiologie, Biostatistiques et Recherche Clinique, Ecole de Santé Publique, Université libre de Bruxelles, Brussels, Belgium

[5] Institut de recherche en sciences de la santé (IRSS), Ouagadougou, 03 BP 7102, Burkina Faso

[6] Département de recherche clinique, centre Muraz, Bobo-Dioulasso, Burkina Faso

[7] Institut Africain de Santé publique (IASP), Ouagadougou, 12 BP 199, Burkina Faso

* corresponding author : bruno.bonnechere@uhasselt.be

Prof. Dr. Bonnechère Bruno

Agoralaan Gebouw A

3590 Diepenbeek

Belgium

For peer review only - http://bmjopen.bmj.com/site/about/guidelines.xhtml

Word count: 3396

Tables: 5

Figures: 2

For peer review only

For peer review only - http://bmjopen.bmj.com/site/about/guidelines.xhtml

Abstract

Objectives: Lifestyle modifiable risk factors are a leading preventable cause of non-communicable diseases (NCDs). Amongst them, alcohol and tobacco consumption are the most important. Studies characterizing the prevalence of alcohol consumption in low-income countries are lacking. This study describes the prevalence of different levels of alcohol consumptions in Burkina Faso and its associated factors.

Design: Data from the 2013 Burkina Faso World Health Organization (WHO) Stepwise approach to Surveillance (STEPS), a population-based cross-sectional survey, were analyzed. The prevalence of alcohol consumption over the last 30 days was recorded and recoded into categories according to WHO recommendations: low, mid or heavy alcohol consumption. Multinomial logistic regression analyses identified factors associated with the different levels of alcohol consumption use.

Setting: Population-based cross-sectional survey

Participants: 4692 participants of both sexes aged 25 to 64 years were included in the study.

Results: In the whole sample 3,559 participants (75.8% [72.5 – 78.7]) were not consuming any alcohol, 614 (12.9% [10.9 – 15.3]) had a low alcohol consumption, 399 (8.5% [7.1 – 10.1]) a mid alcohol consumption and 120 (2.7% [2.0 – 3.7]) a heavy consumption. Age was associated with alcohol intake with a gradient effect, older people having a higher risk for the different levels of consumptions. Tobacco consumption was also significantly associated with alcohol intake with a gradient effect, those with higher tobacco consumption being at higher risk of abusive alcohol intake.

For peer review only - http://bmjopen.bmj.com/site/about/guidelines.xhtml

Conclusion: Our data showed an important burden of alcohol consumption in Burkina Faso with large disparities across the different regions. To effectively reduce alcohol consumption in Burkina Faso, comprehensive control and prevention campaigns should consider these associated factors.

Keywords

Alcohol consumption; Abusive alcohol consumption; Burkina Faso; Risk factors; Surveillance; WHO STEPS survey

For peer review only

Article summary

Strengths and Limitations of this study

- Alcohol consumption is one of the most important modifiable factors of non-communicable disease
- Studies characterizing the prevalence of alcohol consumption in low-income countries are lacking
- We presented alcohol consumption level and associated risk factor in Burkina Faso using a large cohort of 4672 participants
- Large disparities were found between the different region of the countries as well as important correlation with tobacco consumption levels.
- Targeted prevention campaigns should be carried out in order to target these double consumptions which are particularly harmful.

For peer review only - http://bmjopen.bmj.com/site/about/guidelines.xhtml

Background

The increase in life expectancy [1] combined with unhealthy behaviors and physical inactivity [2] are linked to a rise in non-communicable diseases (NCDs) such as cardiovascular disease, cancer, chronic respiratory disease, and diabetes [3]. Currently, the leading causes of ill health in the world, NCDs account for seven out of ten deaths worldwide [4].

Low and mid-income countries are facing the emergence of non-communicable diseases (NCDs) [5]. Although some risk factors have been identified such as high blood pressure [6], lack of physical activity [7], inadequate diet [8], etc., there is still a lack of comprehensive data on other modifiable risk factors, in particular tobacco and alcohol consumption [9], in Burkina Faso.

Recently, clear evidence of increased alcohol consumption and attributable harm in many low and mid-income countries has been highlighted [10]. Furthermore, it is predicted to have a more harmful effect if an effective policy is not adopted [11]. Currently, the prevalence of alcohol intake among the African adult population is about 30% [12], which is lower compared to the rest of the world where 40% of the world's adult population consumes alcohol and the average consumption per drinker is 17.1 liters per year. Interestingly, the prevalence of lifetime abstention, the level of alcohol consumption, and the drinking patterns vary widely across regions of the world [13]. For example, Eastern Europe and Southern Africa had the most detrimental pattern of drinking scores. At the same time, Europe (Eastern and Central) and sub-Saharan Africa (Southern and West) are the most important consumers of alcohol [13]. In Africa, among current drinkers, the prevalence of heavy drinking varied between 7%

For peer review only - http://bmjopen.bmj.com/site/about/guidelines.xhtml

and 77%, and the prevalence of daily light drinkers varied between 0% and 21%. Overall, drinking patterns varied significantly between and within the examined African countries [14].

One of the major current concerns related to alcohol consumption in Africa is that alcohol companies have targeted Africa as a new market. With the expected increases in the number of potential new alcohol consumers, especially young people, and women, the African continent has indeed been identified by the alcohol beverage industry and market researchers as a key area for alcohol market growth [15,16]. It is, therefore, of particular importance to identify subjects the most at risk of alcohol consumption. Currently, two major risk factors have been identified worldwide: gender with the male being more at risk [17] and the socioeconomic level where the effect is less clear as the associations between socioeconomic disadvantage and heavier drinking vary depending on country-level income [18]. A group that is particularly at risk because of the vulnerability of the fetus is pregnant women [19–21]. There is currently a lack of information about the prevalence of alcohol consumption in Burkina Faso and the associated risk factors. Differences in cultural factors (e.g., beliefs and practices) may influence health status, but social, economic, and structural determinants of health during people's lifespans appear to be associated with health inequities between ethnic groups as well. Therefore, cultural influences should not be overemphasized as discrete explanatory factors for health inequities [22].

A first national survey was conducted in 2013 using the WHO STEPwise approach to risk factors surveillance (STEPS). The STEPS survey is a simple, standardized method for collecting, analyzing, and disseminating data in WHO member countries. It covered a representative sample of the adult population. The first analyses performed were about the evaluation of cardiovascular risk [23]. In a previous study, we show that tobacco consumption was highly

For peer review only - http://bmjopen.bmj.com/site/about/guidelines.xhtml

correlated with alcohol consumption in men [24]. Therefore, this study aims to study the prevalence of alcohol consumption and its associated risk factors. Such kind of analysis is needed to drive more efficient prevention campaigns for both alcohol and tobacco consumption.

Methods

Study settings

Burkina Faso is a landlocked country in West Africa of 272 967.47 km². The country is divided into 13 administrative regions and is limited in the North and West by Mali, in the East by Niger, and in the South by Benin, Togo, Ghana and Côte d'Ivoire.

In 2020, the population was estimated at 21,510,181 inhabitants [25]. The majority of the population (77.30%) lives in rural areas and relies on agriculture and livestock as the main source of income [26].

The median age of the population is 17.9 years old. In 2018, the total fertility rate was 5.2 and the life expectancy at birth was 62.3 years in 2019 [27]. As most of the low-income countries, Burkina Faso must face the double burden of infectious and chronic diseases. The country is regularly confronted with outbreaks such as measles, cerebrospinal meningitis and, malaria [28], while NCDs constitute a rising public health problem with only limited financial resources allocated.

In Burkina Faso, several sources of alcohol manufacturing exist modern brewery and legally imported alcohol, prohibited alcohol mostly imported from neighboring countries and traditional beer manufacturers. Burkina Faso is a secular country with more than half of the

For peer review only - http://bmjopen.bmj.com/site/about/guidelines.xhtml

population declared as being of Islamic faith (61.6%), 22.5% Christians, and 15.4% traditional religions [29].

Harmful alcohol use and its adverse events (i.e., road traffic accidents, domestic violence) are however important and raising public health concerns in Burkina Faso [30].

Study design

This is an analytical cross-sectional study of the WHO STEPS survey conducted in Burkina Faso in 2013. Complete details on the methodology and sampling procedure were described in the WHO STEPS 2013 Burkina report [31], and in previous studies using this database [23,24,32]. The participants were selected using a three-stage cluster sampling process described in the sampling method.

Study population

Participants of the study were adults of both sexes aged 25 to 64 years old who had been living in Burkina Faso for at least six months. Exclusion criteria were: people with disabilities hampering their ability to answer the questions (e.g., intellectual disabilities, serious mental disorders, cognitive impairment, etc.).

Patient and public involvement

No patients were involved in the study design or analysis of the anonymous data set.

Sampling

The STEPS survey of Burkina Faso was conducted on a representative sample of 4,800 individuals, the response rate of the STEPS survey was 99.1% in Burkina Faso [28]. After

For peer review only - http://bmjopen.bmj.com/site/about/guidelines.xhtml

excluding participants with missing information about sampling weight, 4,692 individuals were included in the present analysis. The diagram flow of study participants is presented in Figure 1. A three levels sampling frame was performed. The first level of the selection is based on enumerations areas (clusters) in each stratum (urban and rural), uniform sample of households was then selected in each cluster. In the last stage one single participant was randomly selected from all eligible listed household members using the Kish method.

Data collection

A full description of the study design and the data collection has been published elsewhere [23,32]. Briefly, all data on alcohol consumption were collected using a standardized questionnaire during face-to-face interviews in the language most spoken by the respondent. The questionnaires was pre-tested on the field before being used for the national survey. Alcohol consumption was assessed with the question 'Have you ever consumed any alcohol such as beer, wine, spirits or dolo'? If the answer is yes, the current alcohol consumption was assessed with the questions 'During the past 30 days, how many days did you consume alcohol drinks?' and 'When you are drinking, what is the average number of drinks ?'. Four levels were defined: no consumption, low, mid, and abusive consumption (see Table 1 for WHO definition). Other information such as demographic information, anthropometric and behavioral measurements were also collected.

Study variables

The dependent variable was the mean alcohol consumption in the last 30 days. First, we analyzed alcohol consumption to define the profile of the drinker.

For peer review only

The independent variables were: age, sex, education, marital status, occupational status and tobacco consumption (see Table 1 for the definition of the variables).

Data analysis and processing

Some independent quantitative variables were transformed into categorical variables. The list of the variables with the recoded variables is presented in Table 1. Qualitative variables were described using proportions. The Chi-squared test was used in univariable analysis to test the association between the outcome and the categorical variables. Multivariable analysis was then performed: we used a logistic regression model fitted using stepwise backward regression modeling. We first analyzed the profile and levels of alcohol consumption using multinomial logistic regression. Then we identify the profile of the abusive consumer, following WHO recommendations [33], amongst current drinkers using logistic regression grouping low and mid consumption as reference.

Adjusted Odds Ratios (ORs) were calculated for the studied variables and presented with 95% confidence intervals. We then analyzed discrepancies between the different regions using adjusted ORs for the different regions.

All the analyses were carried out using the survey function with the sampling weight option to account for the sampling design.

Statistics have been conducted in STATA (version 13), RStudio (version 1.1.442) with R version 3.4.4).

For peer review only - http://bmjopen.bmj.com/site/about/guidelines.xhtml

Results

Characteristics of the population are presented in Table 2. Most of the participants were rural residents (79.7%). The population was predominantly young, with the 25-34 years group representing 41.9%. Women were a bit more represented, with 54.3% of the total sample. Concerning the comparison between urban and rural residents there is no difference in term of age ($\chi^2(3) = 2.36$, $p = .51$), sex ($\chi^2(1) = 3.71$, $p = .054$), but there are statistically significant differences for the educational level ($\chi^2(2) = 924$, $p < .001$), the marital status ($\chi^2(2) = 233$, $p < .001$), the occupational status ($\chi^2(3) = 810$, $p <.001$).

In the whole population 3,559 (75.8% [72.5 – 78.7]) were not consuming any alcohol, 614 (12.9% [10.9 – 15.3]) had a low alcohol consumption, 399 (8.5% [7.1 – 10.1]) a mid alcohol consumption and 120 (2.7% [2.0 – 3.7]) an abusive consumption. There are important differences between the different regions of the countries; results are presented in Figure 2. In the *Sahel* region the prevalence is null while in the Sud-Ouest regions the prevalence for all the levels are statistically significantly higher than the country's average values.

The prevalence of abusive consumption was not different in urban setting and rural setting (4.3 % [2.5 – 7.2] versus 2.1% [1.5 – 3.1], p = .19). The repartition of consumption was statistically significantly different between urban and rural environment (p =.001): There were a higher proportion of low alcohol consumption is rural area (13.1% compared to 12.7%) while in urban area there is more mid (10.0% Vs. 8.0%) and high consumption (4.3% Vs. 2.2%).

In multivariable analysis (Table 3), first concerning the alcohol consumption, there is a statistically significant gradient effect of the age regardless the level of consumption (AOR = 1.46 [1.09 – 1.95], 1.79 [1.34 – 2.40], and 2.36 [1.59 – 3.51] for the 35-44, 45-54 and 55-64

For peer review only - http://bmjopen.bmj.com/site/about/guidelines.xhtml

years old respectively compared to the 25-34 years old group for low consumption, AOR = 1.55 [1.14 – 2.12], 1.81 [1.25 – 2.62], and 2.50 [1.54 – 4.07] for mid consumption and AOR = 1.86 [1.06 – 3.27], 2.67 [1.36 – 5.25], 2.37 [1.01 – 5.92] for abusive consumption). Interestingly, the effect of age is different regarding the level of consumption: there is an increased risk in male for low (AOR = 2.68 [1.96 – 3.67]) and abusive consumption (AOR = 2.89 [1.56 – 2.37]), while there is a protective effect against moderate consumption (AOR = 0.38 [0.26 – 0.55]). The level of education is also associated with an increased risk, regardless of consumption level (AOR = 2.08 [1.07 – 4.03], 2.97 [1.48 – 5.92], and 3.50 [1.34 – 9.15] for low, mid, and abusive consumption respectively for participants who reached secondary levels in comparison of people without eduction).

There is also an important gradient effect associated with tobacco consumption: the consumption of small tobacco quantity (<5 cigs/day) is associated with an increased risk of low alcohol consumption (AOR = 1.72 [1.13 – 2.62]) while the consumption of high quantity of tobacco is associated with mid and abusive alcohol consumption (AOR = 6.08 [2.75 – 13.4] and 6.58 [1.96 – 22.11] respectively).

Finally, we computed the risk of having abusive consumption in those consuming alcohol (Table 4). Sex is an important risk factor with increased risk for the men compared to women (AOR = 2.53 [1.38 – 4.68]) as well as the occupational status with jobless people and housemaker associated with a decreased risk of having abusive consumption (AOR = 0.05 [0.01 – 0.47] and AOR = 0.14 [0.02 – 0.78] respectively).

The risk also varied from the different regions, AORs for the different regions of the countries are presented in Table 5. We observed that the *Sud-Ouest* region has a higher risk of

For peer review only

For peer review only - http://bmjopen.bmj.com/site/about/guidelines.xhtml

consumption compared to the rest of the country. In the *Sahel*, *Est* and *Haut-Bassin*, the risks of consumption are significantly lower.

Discussion

We report here the results of the first nationally representative survey on the prevalence and risk factors for alcohol consumption in Burkina Faso.

The overall prevalence of people with abusive alcohol consumption is 2.7% [2.0 – 3.7]. This consumption is lower compared to other African countries [12] and the rest of the world [13]. However, this prevalence might be underestimated because even though Burkina Faso is a secular country, the majority of its inhabitants have a religious faith that prohibits alcohol consumption that could prevent some people from declaring their alcohol intake, this might be particularly true in the *Sahel* region. Also, alcohol consumption is blamed by society and could also lead to an under-declaration (positive social perception bias) [34].

We also found differences in the different regions of the country with the highest levels of consumption found in the *Sud-Ouest* region. In this region, almost every household produces local beer ('*dolo*') this might explain this observation. The income disparities and the alcohol availability between the different regions could also explain those differences [35]. From a public health perspective, this region also has the highest prevalence of Hepatitis B and C [36]. The inhabitants of these regions seem to cumulate the hepatotoxic risk of Hepatitis and alcohol consumption. We found a null prevalence in the Sahel region, this is an entirely Muslim region and the desirability bias may be more important compared to other regions.

We also found gender differences with an increased risk in men compared to women for low and abusive alcohol consumption but an increased risk of moderate consumption for women.

For peer review only - http://bmjopen.bmj.com/site/about/guidelines.xhtml

It is important to specify here that, following the WHO guidelines, the thresholds used to define the different levels of alcohol consumption are different for men and women (33 to 50% lower for women compared to men). The risk of abusive consumption in drinkers is more important in men compared to women.

In most countries, the prevalence is higher in men compared to women [17,37], but when adjusting for multiple factors such as social supports and financial aspects, this effect seems less important. Compared to men, more women are lifetime abstainers, drink less, and are less likely to engage in problem drinking. Of note is that women drinking excessively develop more severe medical problems than man. Biological (sex-related) factors, including differences in alcohol pharmacokinetics as well as its effect on brain function and the levels of sex hormones, may contribute to some of those differences [38]. Since pregnant women are particularly vulnerable, we performed a sub-group analysis. A subgroup of 299 out of the 2,449 (12.2%) women was pregnant during the survey, amongst them, 6 (3.9%) reported alcohol consumption, which is statistically lower compared to non-pregnant women (12.6%, p = .001).

Interestingly we observed that the age was significantly associated with alcohol consumption with a gradient effect of age on alcohol consumption, but it seems that the level of consumption is not influenced by gender as the OR are relatively similar for the different categories. The influence of age on alcohol consumption is still unclear and not well documented in the literature, except for binge drinking, where young adults are the most at risk [39]. However, this result should be interpreted carefully, considering our study design. This could be the results of preventive campaigns as reflected by a cohort effect A large Australian study including 7 cross-sectional waves showed indeed that male cohorts born

For peer review only - http://bmjopen.bmj.com/site/about/guidelines.xhtml

between 1965 and 1974 and female cohorts born between 1955 and 1974 reported higher rates of drinking participation ($P < 0.05$), while the most recent cohorts (born in the 1990s) had lower rates of participation ($P < 0.01$) [40].

Concerning the risk of switching to an abusive consumption among drinkers only two factors have been identified: the risk in increased in male (as for the general consumption) and decreased with the occupational status, probably due to financial constraints.

The association with tobacco consumption is probably one of the most important from a public health view because of the comorbidities and the double burden it presents for the population. In a previous study, we identified people most at risk of tobacco consumption in Burkina Faso: tobacco smoking among men was significantly associated with increased age and alcohol consumption. Analysis of risk factors for other tobacco use stratified by gender shows that age, education, residence, and alcohol consumption were significantly associated with consumption for women, age, and alcohol consumption for men [24].

As recommended by the WHO, in this paper we presented risk factors associated with abusive alcohol consumption because of the proven negative effects. It is, however, interesting to note that from a medical point of view a limited and reasonable alcohol consumption could have some health-related benefits: for example, the relation between dementia and cognitive disorders is not linear and limited alcohol consumption has a protective role for dementia [41–43] or the protective effect of alcohol on cardiovascular risk, previously known as *'the French paradox'* [44–46]. However, these results must be interpreted with caution, especially since it is known that even in low doses, alcohol consumption transiently increases the risk of cardiovascular accidents [47]. Another important point is that considering the prevention side experiences showed that adopting a too strong position by prohibiting any consumption or

For peer review only

For peer review only - http://bmjopen.bmj.com/site/about/guidelines.xhtml

behavior will lead to poor results [48,49]. Considering these two aspects low alcohol consumption could be considered as acceptable. Alcohol policies that regulate the physical availability of alcohol are associated with lower alcohol consumption in low- and middle-income countries [50]. Burkina Faso is at an embryonic stage in this area. Indeed, the country just had its first specifications plan on alcoholic beverages production, importation, and selling adopted in 2020 [51]. This plan was jointly approved by the Ministry of Trade and the companies involved in such activities. Some additional measurements have been adopted by the government. These include the prohibition of advertisements for tobacco and alcohol products and, since September 19, 2019 the government also banned the production, importation and marketing of liqueurs and other spirit drinks in plastic bags and in bottles of less than 30 centiliters to limit the availability [52]. This plan needs to be rigorously implemented first and then rapidly incremented to a stronger legislative law in order to positively impact behavior and health outcomes.

The main limitation of this study is that alcohol (and tobacco) consumption were obtained during interviews and is therefore dependent on the faith of the participants. There is, therefore, both a risk of memory bias and social desirability, probably more marked during pregnancy. It can thus be estimated that the numbers and prevalence obtained in this survey underestimate the actual consumption. Another potential limitation is that some well-known risk factors for alcohol consumption were not included in the study because data on these variables have not been collected during the STEPS survey. Part of such variables is socioeconomic status. It is a transversal study; therefore there is a risk of survival bias indicating that older participants with high consumption may die prematurely due to this consumption [53].

For peer review only - http://bmjopen.bmj.com/site/about/guidelines.xhtml

Despite these limitations, given the study design (cluster sampling design) and the sample size, the results of this study can be extended to the whole of Burkina Faso.

Conclusion

In this study, we reported on the burden of alcohol consumption and associated risk factors in a nationally representative sample of adults in Burkina Faso. Our data showed an important burden of alcohol consumption in Burkina Faso. Tobacco consumption is an important modifiable risk factor associated with alcohol consumption. Zone-specific interventions are needed given the higher burden in urban centers and some specific regions such as the *Sud-Ouest* region. Health policies in Burkina Faso must henceforth account for the control of alcohol and tobacco consumption since there is a strong relationship between those two important risk factors of NCDs. Alcohol policies that regulate the physical availability of alcohol are associated with lower alcohol consumption and should, therefore, be implemented in Burkina Faso to reduce the burden of alcohol consumption.

List of abbreviation

DHS: Demographic and Health Surveys

NCD: Non-communicable disease

SSA: sub-Saharan Africa

STEPS: WHO STEPwise approach to surveillance

WHO: World Health Organization

For peer review only - http://bmjopen.bmj.com/site/about/guidelines.xhtml

Declarations

Ethics approval and consent to participate Consent for publication

The protocol of the STEPS survey was reviewed and approved by the Ethics Committee for Health Research of the Ministry of Health of Burkina Faso, which gave clearance in accordance with regulations in force (Deliberation No. 2012–12- 092 of 05 December 2012). Written informed consent was systematically sought and obtained from all participants before inclusion in the study. The confidentiality of study participants was fully respected and the analyses performed did not identify any participant. For this study, we obtained authorization from the General Directorate of Health to reanalyze the data, and the confidentiality of study participants was preserved.

Availability of data and materials

The dataset of the STEPS survey that was used in this research is available at the Ministry of Health upon request. Other STEPS surveys can be obtained through the WHO (https://www.who.int/ncds/surveillance/steps/riskfactor/en/). Any request to reanalyze the data can be directed to Dr Brice Bicaba (bicababrico78@gmail.com). No further additional data are available.

Acknowledgments

The authors are thankful to all the people involved in the STEPS survey. Bruno Bonnechère thanks people who help him during the analysis of the data in Burkina Faso.

For peer review only

Competing interests

The authors declare that they have no competing interests.

Funding

Bonnechère Bruno received a travel grant from ARES (https://www.ares-ac.be/fr/) to conduct the data analysis in Burkina Faso (second analysis of the STEP survey), interpret the data with local epidemiologists and write the manuscript. Kadari Cissé is funded by ARES. The funding body had no role in study design, data collection and analysis, decision to publish, or preparation of the manuscript

Author contributions

BB and FKS conceived the study. BB performed the analysis. BB, SS and CK proposed an early draft of the paper. FKS, SK, and ST made substantial contributions to the conception and design, analysis and interpretation of the data. KFS, SK and ST contributed significantly to revise the manuscript. All authors read and approved the final manuscript.

For peer review only

For peer review only - http://bmjopen.bmj.com/site/about/guidelines.xhtml

Figures captions

Figure 1: Consort Diagram describing study participants, exclusions and analytic sample size.

Figure 2 : Prevalence of the level of alcohol consumption in the different regions of Burkina Faso. Vertical lines indicate the mean prevalence for the whole country.

For peer review only

For peer review only - http://bmjopen.bmj.com/site/about/guidelines.xhtml

References

1 GBD 2015 DALYs and HALE Collaborators. Global, regional, and national disability-adjusted life-years (DALYs) for 315 diseases and injuries and healthy life expectancy (HALE), 1990-2015: a systematic analysis for the Global Burden of Disease Study 2015. *Lancet Lond Engl* 2016;**388**:1603–58. doi:10.1016/S0140-6736(16)31460-X

2 Lee I-M, Shiroma EJ, Lobelo F, *et al.* Effect of physical inactivity on major non-communicable diseases worldwide: an analysis of burden of disease and life expectancy. *Lancet Lond Engl* 2012;**380**:219–29. doi:10.1016/S0140-6736(12)61031-9

3 NCD Countdown 2030 collaborators. NCD Countdown 2030: worldwide trends in non-communicable disease mortality and progress towards Sustainable Development Goal target 3.4. *Lancet Lond Engl* 2018;**392**:1072–88. doi:10.1016/S0140-6736(18)31992-5

4 World Health Organization. World Health Statistics 2018: Monitoring health for the SDGs. 2018.

5 Jamison DT, Summers LH, Alleyne G, *et al.* Global health 2035: a world converging within a generation. *Lancet Lond Engl* 2013;**382**:1898–955. doi:10.1016/S0140-6736(13)62105-4

6 Kearney PM, Whelton M, Reynolds K, *et al.* Global burden of hypertension: analysis of worldwide data. *Lancet Lond Engl* 2005;**365**:217–23. doi:10.1016/S0140-6736(05)17741-1

7 Whiteford HA, Degenhardt L, Rehm J, *et al.* Global burden of disease attributable to mental and substance use disorders: findings from the Global Burden of Disease Study 2010. *Lancet Lond Engl* 2013;**382**:1575–86. doi:10.1016/S0140-6736(13)61611-6

8 Afshin A, Micha R, Webb M, *et al.* Effectiveness of Dietary Policies to Reduce Noncommunicable Diseases. In: Prabhakaran D, Anand S, Gaziano TA, *et al.*, eds. *Cardiovascular, Respiratory, and Related Disorders*. Washington (DC): : The International Bank for Reconstruction and Development / The World Bank 2017. http://www.ncbi.nlm.nih.gov/books/NBK525147/ (accessed 10 Sep 2019).

9 Juma PA, Mohamed SF, Matanje Mwagomba BL, *et al.* Non-communicable disease prevention policy process in five African countries. *BMC Public Health* 2018;**18**:961. doi:10.1186/s12889-018-5825-7

10 Shield K, Manthey J, Rylett M, *et al.* National, regional, and global burdens of disease from 2000 to 2016 attributable to alcohol use: a comparative risk assessment study. *Lancet Public Health* 2020;**5**:e51–61. doi:10.1016/S2468-2667(19)30231-2

11 Manthey J, Shield KD, Rylett M, *et al.* Global alcohol exposure between 1990 and 2017 and forecasts until 2030: a modelling study. *The Lancet* 2019;**393**:2493–502. doi:10.1016/S0140-6736(18)32744-2

For peer review only - http://bmjopen.bmj.com/site/about/guidelines.xhtml

12 World Health Organization. Global Status Report on Alcohol and Health—2014. 2014.

13 Shield KD, Rylett M, Gmel G, *et al.* Global alcohol exposure estimates by country, territory and region for 2005--a contribution to the Comparative Risk Assessment for the 2010 Global Burden of Disease Study. *Addict Abingdon Engl* 2013;**108**:912–22. doi:10.1111/add.12112

14 Clausen T, Rossow I, Naidoo N, *et al.* Diverse alcohol drinking patterns in 20 African countries. *Addict Abingdon Engl* 2009;**104**:1147–54. doi:10.1111/j.1360-0443.2009.02559.x

15 Reuters. SABMiller Eyes Africa $2.5bn Investment. 2012.http://www.iol.co.za/business/international/sabmiller-eyes-africa-25bn-investment-1277086 (accessed 8 Jul 2019).

16 African Business. Alcohol Consumption Rising in Africa Despite Obstacles. https://africanbusinessmagazine.com/sectors/retail/alcohol-consumption-rising-in-africa-despite-obstacles/ (accessed 8 Jul 2019).

17 Chaiyasong S, Huckle T, Mackintosh A-M, *et al.* Drinking patterns vary by gender, age and country-level income: Cross-country analysis of the International Alcohol Control Study. *Drug Alcohol Rev* 2018;**37 Suppl 2**:S53–62. doi:10.1111/dar.12820

18 Huckle T, Romeo JS, Wall M, *et al.* Socio-economic disadvantage is associated with heavier drinking in high but not middle-income countries participating in the International Alcohol Control Study. *Drug Alcohol Rev* 2018;**37 Suppl 2**:S63–71. doi:10.1111/dar.12810

19 Martinez P, Røislien J, Naidoo N, *et al.* Alcohol abstinence and drinking among African women: data from the World Health Surveys. *BMC Public Health* 2011;**11**:160. doi:10.1186/1471-2458-11-160

20 Popova S, Lange S, Probst C, *et al.* Actual and predicted prevalence of alcohol consumption during pregnancy in the WHO African Region. *Trop Med Int Health TM IH* 2016;**21**:1209–39. doi:10.1111/tmi.12755

21 Sanou AS, Diallo AH, Holding P, *et al.* Maternal alcohol consumption during pregnancy and child's cognitive performance at 6-8 years of age in rural Burkina Faso: an observational study. *PeerJ* 2017;**5**:e3507. doi:10.7717/peerj.3507

22 Mosdøl A, Lidal IB, Straumann GH, *et al.* Targeted mass media interventions promoting healthy behaviours to reduce risk of non-communicable diseases in adult, ethnic minorities. *Cochrane Database Syst Rev* 2017;**2**:CD011683. doi:10.1002/14651858.CD011683.pub2

23 Soubeiga JK, Millogo T, Bicaba BW, *et al.* Prevalence and factors associated with hypertension in Burkina Faso: a countrywide cross-sectional study. *BMC Public Health* 2017;**17**:64. doi:10.1186/s12889-016-3926-8

For peer review only

24 Bonnechère B, Cissé K, Millogo Tiéba, *et al.* Tobacco use and associated risk factors in Burkina Faso: Results from a nationwide population-based survey. *BMC Public Health* 2019.

25 Ministère de l'économie et des finances. Projection démographique de 2007 à 2020 par région et province. Institut national de la statistique et de la démographie, Burkina Faso. http://www.cns.bf/IMG/pdf/projections_demographiques_sous_nationales.pdf

26 Institut National de la Statistique et de la Démographie (INSD); ICF International. Enquête Démographique et de Santé et à Indicateurs Multiples du Burkina Faso 2010. 2012.

27 Institut national de la statistique et de la démographie. BURKINA FASO MINISTERE DE L'ECONOMIE ET DES FINANCES: PROJECTIONS DEMOGRAPHIQUES DE 2007 A 2020 PAR REGION ET PROVINCE. http://www.cns.bf/IMG/pdf/projections_demographiques_sous_nationales.pdf

28 Ministère de la santé du Burkina Faso. Rapport de l'enquête nationale sur la prévalence des principaux facteurs de risque communs aux maladies non transmissibles (STEPS 2013). 2014.

29 La Croix A. Données géographiques et identité religieuse au Burkina Faso. Données Géographiques Identité Relig. Au Burkina Faso. https://africa.la-croix.com/statistiques/burkina-faso/ (accessed 28 Feb 2021).

30 Bonnet E, Lechat L, Ridde V. What interventions are required to reduce road traffic injuries in Africa? A scoping review of the literature. *PloS One* 2018;**13**:e0208195. doi:10.1371/journal.pone.0208195

31 WHO. STEPwise approach to chronic disease risk factor surveillance. https://www.who.int/ncds/surveillance/steps/burkina_faso/en/

32 Millogo T, Bicaba BW, Soubeiga JK, *et al.* Diabetes and abnormal glucose regulation in the adult population of Burkina Faso: prevalence and predictors. *BMC Public Health* 2018;**18**. doi:10.1186/s12889-018-5257-4

33 Kendagor A, Gathecha G, Ntakuka MW, *et al.* Prevalence and determinants of heavy episodic drinking among adults in Kenya: analysis of the STEPwise survey, 2015. *BMC Public Health* 2018;**18**:1216. doi:10.1186/s12889-018-6057-6

34 Jemmott JB, Ditto PH, Croyle RT. Judging health status: effects of perceived prevalence and personal relevance. *J Pers Soc Psychol* 1986;**50**:899–905. doi:10.1037//0022-3514.50.5.899

35 Dixon MA, Chartier KG. Alcohol Use Patterns Among Urban and Rural Residents: Demographic and Social Influences. *Alcohol Res Curr Rev* 2016;**38**:69–77.

36 Meda N, Tuaillon E, Kania D, *et al.* Hepatitis B and C virus seroprevalence, Burkina Faso: a cross-sectional study. *Bull World Health Organ* 2018;**96**:750–9. doi:10.2471/BLT.18.208603

For peer review only - http://bmjopen.bmj.com/site/about/guidelines.xhtml

37 Takahashi R, Wilunda C, Magutah K, *et al.* Correlates of alcohol consumption in rural western Kenya: A cross-sectional study. *BMC Psychiatry* 2017;**17**:175. doi:10.1186/s12888-017-1344-9

38 Erol A, Karpyak VM. Sex and gender-related differences in alcohol use and its consequences: Contemporary knowledge and future research considerations. *Drug Alcohol Depend* 2015;**156**:1–13. doi:10.1016/j.drugalcdep.2015.08.023

39 Kuntsche E, Kuntsche S, Thrul J, *et al.* Binge drinking: Health impact, prevalence, correlates and interventions. *Psychol Health* 2017;**32**:976–1017. doi:10.1080/08870446.2017.1325889

40 Livingston M, Raninen J, Slade T, *et al.* Understanding trends in Australian alcohol consumption-an age-period-cohort model. *Addict Abingdon Engl* 2016;**111**:1590–8. doi:10.1111/add.13396

41 Heffernan M, Mather KA, Xu J, *et al.* Alcohol Consumption and Incident Dementia: Evidence from the Sydney Memory and Ageing Study. *J Alzheimers Dis JAD* 2016;**52**:529–38. doi:10.3233/JAD-150537

42 Xu W, Wang H, Wan Y, *et al.* Alcohol consumption and dementia risk: a dose-response meta-analysis of prospective studies. *Eur J Epidemiol* 2017;**32**:31–42. doi:10.1007/s10654-017-0225-3

43 Gutwinski S, Schreiter S, Priller J, *et al.* Drink and Think: Impact of Alcohol on Cognitive Functions and Dementia - Evidence of Dose-Related Effects. *Pharmacopsychiatry* 2018;**51**:136–43. doi:10.1055/s-0043-118664

44 O'Keefe JH, Bhatti SK, Bajwa A, *et al.* Alcohol and cardiovascular health: the dose makes the poison…or the remedy. *Mayo Clin Proc* 2014;**89**:382–93. doi:10.1016/j.mayocp.2013.11.005

45 Fernández-Solà J. Cardiovascular risks and benefits of moderate and heavy alcohol consumption. *Nat Rev Cardiol* 2015;**12**:576–87. doi:10.1038/nrcardio.2015.91

46 Rehm J, Roerecke M. Cardiovascular effects of alcohol consumption. *Trends Cardiovasc Med* 2017;**27**:534–8. doi:10.1016/j.tcm.2017.06.002

47 Mostofsky E, Chahal HS, Mukamal KJ, *et al.* Alcohol and Immediate Risk of Cardiovascular Events: A Systematic Review and Dose-Response Meta-Analysis. *Circulation* 2016;**133**:979–87. doi:10.1161/CIRCULATIONAHA.115.019743

48 Cyril S, Smith BJ, Renzaho AMN. Systematic review of empowerment measures in health promotion. *Health Promot Int* 2016;**31**:809–26. doi:10.1093/heapro/dav059

49 Gelius P, Rütten A. Conceptualizing structural change in health promotion: why we still need to know more about theory. *Health Promot Int* 2018;**33**:657–64. doi:10.1093/heapro/dax006

For peer review only

For peer review only - http://bmjopen.bmj.com/site/about/guidelines.xhtml

50 Cook WK, Bond J, Greenfield TK. Are alcohol policies associated with alcohol consumption in low- and middle-income countries? *Addict Abingdon Engl* 2014;**109**:1081–90. doi:10.1111/add.12571

51 Ministère de l'Industrie, du Commerce et de l'Artisanat. Communiqué MICA. https://www.commerce.gov.bf/accueil (accessed 3 Mar 2021).

52 Burkina24. Production et commercialisation d'alcool au Burkina : Bientôt un cahier de charges. https://www.burkina24.com/2020/07/02/production-et-commercialisation-dalcool-au-burkina-bientot-un-cahier-de-charges/

53 Roerecke M. On bias in alcohol epidemiology and the search for the perfect study. *Addict Abingdon Engl* 2017;**112**:217–8. doi:10.1111/add.13549

For peer review only

For peer review only - http://bmjopen.bmj.com/site/about/guidelines.xhtml

Tables

Table 1: Definition of recoded exposure variables.

Variables	Categories
Age groups	"25 to 34 years old", "35 to 44 years old", "45 to 54 years old", "55 to 64 years old"
Education	"None", "Primary", "Secondary", "Tertiary"
Marital status	"Single", "Married", "Divorced/widowed"
Occupational status	"Wage earner", "Self-employed", "Jobless", "Housemaker"
Alcohol consumption (WHO recommendations) [30]	None: Never intake of alcohol<br>Low: intake of an average quantity of pure alcohol of less than 40 g per day for men and less than 20 g for women<br>Mid: corresponds to taking an average quantity of pure alcohol of between 40 g and 59.9 g per day for men and between 20 g and 39.9 g for women<br>Abusive: intake of an average quantity of pure alcohol greater than or equal to 60 g per day for men and greater than or equal to 40 g for women.

Note than one glass of alcohol (beer, wine, 'dolo') contains 10g of ethanol, the recall period is 30 days.

For peer review only

For peer review only - http://bmjopen.bmj.com/site/about/guidelines.xhtml

Table 2: Socio-demographic characteristics of the study sample and prevalence of alcohol and abusive alcohol consumption

Variables	Participants, n (%)	Low consumption [95% CI]	Mid consumption [95% CI]	Abusive consumption, % [95% CI]
**Age groups, years (n= 4,692)**				
25 to 34 years	2,124 (42.0)	10.6 [8.4 – 13.1]	7.0 [5.5 – 8.8]	1.9 [1.2 – 2.9]
35 to 44 years	1,181 (27.9)	13.1 [10.4 – 16.3]	8.9 [6.8 – 11.5]	3.1 [1.9 – 4.9]
45 to 54 years	841 (18.6)	14.8 [11.0 – 19.6]	9.5 [7.1 – 12.5]	3.8 [2.3 – 6.1]
55 to 64 years	546 (11.5)	18.3 [14.1 – 23.4]	11.3 [8.3 – 15.3]	3.0 [1.5 – 6.2]
**Sex (n = 4,692)**				
Female	2,436 (54.3)	8.6 [6.3 – 11.7]	10.8 [8.8 – 13.3]	0.9 [0.6 – 1.5]
Male	2,256 (45.7)	18.1 [15.5 – 21.1]	5.8 [4.6 – 7.1]	4.8 [3.4 – 6.8]
**Living environment (n = 4,692)**				
Urban	945 (24.9)	12.7 [8.4 – 18.6]	10.0 [7.3 – 13.5]	4.3 [2.5 – 7.2]
Rural	3,747 (75.1)	13.1 [10.8 – 15.6]	8.0 [6.4 – 9.9]	2.2 [1.5 – 3.1]
**Education (n = 4,684)**				
None	3,622 (77.3)	12.2 [9.9 – 14.8]	7.8 [6.2 – 9.7]	2.0 [1.4 – 2.9]
Primary	728 (15.3)	15.6 [11.9 – 20.1]	8.8 [6.7 – 11.5]	3.6 [2.0 – 6.3]
Secondary	334 (7.3)	15.7 [11.7 – 20.7]	15.3 [11.2 – 20.4]	8.2 [4.5 – 14.6]
**Marital status (n = 4,688)**				
Single	333 (6.8)	16.9 [11.8 – 23.6]	9.2 [6.1 – 13.8]	5.2 [2.6 – 10.1]
Married	4,043 (87.2)	12.8 [10.6 – 15.4]	8.2 [6.7 – 9.9]	2.4 [1.8 – 3.3]
Divorced/Widowed	311 (5.9)	10.4 [6.9 – 9.9]	12.5 [8.8 – 17.6]	3.8 [1.6 – 8.5]
**Occupational status (n = 4,692)**				

For peer review only - http://bmjopen.bmj.com/site/about/guidelines.xhtml

Wage earner	281 (6.3)	15.1 [10.7 – 20.9]	12.7 [8.5 – 18.5]	9.9 [5.8 – 16.2]
Self-employed	3,249 (66.4)	13.2 [11.2 – 15.5]	7.9 [6.3 – 9.8]	2.9 [2.1 – 4.3]
Jobless	125 (2.9)	11.8 [6.7 – 20.0]	10.4 [5.7 – 18.2]	0.5 [0.1 – 3.4]
Housemaker	1,037 (24.5)	11.9 [7.6 – 18.2]	8.9 [6.8 – 11.6]	0.4 [0.1 – 1.4]
**Tobacco consumption (n = 4,692)**				
No	4,217 (90.8)	12.1 [10.0 – 14.6]	8.4 [6.9 – 10.1]	2.1 [1.6 – 2.9]
< 5 cigs/day	210 (3.8)	23.9 [17.6 – 31.5]	1.9 [0.8 – 4.5]	6.4 [3.2 – 12.6]
5 – 10 cigs/day	184 (3.6)	19.8 [13.5 – 28.1]	13.8 [8.5 – 21.4]	6.6 [3.2 – 13.0]
> 10 cigs/day	81 (1.7)	18.4 [10.7 – 29.8]	17.9 [9.8 – 30.1]	15.5 [4.6 – 40.7]

For peer review only

For peer review only - http://bmjopen.bmj.com/site/about/guidelines.xhtml

Table 3: Factors associated with alcohol and abusive alcohol consumptions in population aged 25 – 64 years.

Variables	Low consumption		Mid consumption		Abusive consumption	
	n	AOR [95% CI]	n	AOR [95% CI]	n	AOR [95% CI]
**Age groups**						
25 years to 34 years (n = 2,224)	221	Ref	151	Ref	41	Ref
35 years to 44 years (n = 1,181)	161	**1.46 [1.09 – 1.95]***	112	**1.55 [1.14 – 2.12]****	30	**1.86 [1.06 – 3.27]***
44 years to 54 years (n = 841)	123	**1.79 [1.34 – 2.40]*****	81	**1.81 [1.25 – 2.62]****	30	**2.67 [1.36 – 5.25]****
55 to 64 (n =546)	105	**2.36 [1.59 – 3.51]*****	55	**2.50 [1.54 – 4.07]*****	19	**2.37 [1.01 – 5.92]***
**Sex**						
Female (n = 2,436)	204	Ref	270	Ref	22	Ref
Male (n = 2,256)	406	**2.68 [1.96 – 3.67]*****	129	**0.38 [0.26 – 0.55]*****	98	**2.89 [1.56 – 2.37]****
**Living environment**						
Urban (n = 945)	111	Ref	109	Ref	30	Ref
Rural (n = 3,747)	499	1.18 [0.59 – 2.33]	290	1.12 [0.68 – 1.87]	90	0.76 [0.40 – 1.45]

For peer review only - http://bmjopen.bmj.com/site/about/guidelines.xhtml

**Education**						
None (n = 3,622)	452	Ref	281	Ref	81	Ref
Primary (n = 728)	112	**1.47 [1.05 – 2.06]***	67	1.47 [0.99 – 2.18]	19	1.60 [0.81 – 3.18]
Secondary (n = 334)	46	**2.08 [1.07 – 4.03]***	49	**2.97 [1.48 – 5.92]****	19	**3.50 [1.34 – 9.15]****
**Marital status**						
Single (n = 333)	57	Ref	31	Ref	17	Ref
Married (n = 4,043)	514	0.66 [0.40 – 1.10]	93	0.74 [0.43 – 1.30]	93	0.56 [0.23 – 1.36]
Divorced/Widowed (n = 311)	39	0.56 [0.29 – 1.08]	10	0.83 [0.42 – 1.63]	10	1.01 [0.31 – 3.33]
**Occupational status**						
Wage earner (n = 281)	47	Ref	35	Ref	20	Ref
Self-employed (n = 3,249)	436	1.10 [0.58 – 2.06]	246	0.90 [0.53 – 1.51]	95	0.65 [0.26 – 1.67]
Jobless (n = 125)	16	0.75 [0.37 – 1.53]	15	0.77 [0.33 – 1.76]	1	**0.05 [0.01 – 0.44]****
House maker (n = 1,037)	111	2.20 [0.95 – 5.08]	103	0.74 [0.41 – 1.33]	4	0.23 [0.04 – 1.15]
**Tobacco consumption**						
No (n = 4,217)	512	Ref	354	Ref	86	Ref

For peer review only - http://bmjopen.bmj.com/site/about/guidelines.xhtml

< 5 cigs/day (n = 210)	47	**1.72 [1.13 – 2.62]***	6	0.52 [0.21 – 1.31]	14	**2.42 [1.07 – 5.63]***
5 – 10 cigs/day (n = 184)	36	1.60 [0.98 – 2.62]	23	**4.20 [2.25 – 7.83]*****	11	**2.48 [1.06 – 5.79]***
> 10 cigs/day (n = 81)	15	1.77 [0.83 – 3.75]	16	**6.08 [2.75 – 13.4]*****	9	**6.58 [1.96 – 22.11]****

* p = 0.05, ** p = 0.01, *** p < 0.001

For peer review only

For peer review only - http://bmjopen.bmj.com/site/about/guidelines.xhtml

Table 4: Factors associated with abusive alcohol consumptions in population aged 25 – 64 years consuming alcohol.

Variables	Alcohol consumption	Abusive alcohol consumption	
	N	N	AOR [95%CI]
**Age groups**			
25 years to 34 years	413	41	Ref
35 years to 44 years	303	30	1.55 (0.84 – 2.86)
44 years to 54 years	234	30	1.91 (0.95 – 3.85)
55 to 64	179	19	0.99 (0.38 – 2.57)
**Sex**			
Female	496	22	Ref
Male	633	98	**2.53 (1.38 – 4.68)****
**Living environment**			
Urban	250	30	Ref
Rural	879	90	0.68 (0.34 – 1.36)
**Education**			
None	814	81	Ref
Primary	198	19	0.89 (0.43 – 1.86)
Secondary	114	19	1.25 (0.48 – 3.25)
**Marital status**			
Single	105	17	Ref
Married	930	93	0.70 (0.32 – 1.53)
Divorced/Widowed	94	10	1.41 (0.45 – 4.46)
**Occupational status**			
Wage earner	102	20	Ref
Self-employed	777	95	0.51 (0.19 – 1.50)
Jobless	32	1	**0.05 (0.01 – 0.47)****
House maker	218	4	**0.14 (0.02 – 0.78)***
**Tobacco consumption**			
No	952	86	Ref
< 5 cigs/day	67	14	1.71 (0.68 – 4.26)

For peer review only - http://bmjopen.bmj.com/site/about/guidelines.xhtml

5 – 10 cigs/day	70	11	1.20 (0.51 – 2.86)
> 10 cigs/day	40	9	2.26 (0.63 – 8.05)

* p = 0.05, ** p = 0.01, *** p < 0.001

For peer review only

For peer review only - http://bmjopen.bmj.com/site/about/guidelines.xhtml

Table 5: Risk of alcohol consumption in the different regions of the country.

Region	N	Alcohol consumption	
		n	AOR (95%CI)
Centre	548	168	Ref
Boucle du Mouhoun	469	147	1.23 [0.63 – 2.39]
Cascades	166	45	0.58 [0.28 – 1.19]
Centre-Est	398	72	0.51 [0.24 – 1.06]
Centre-Nord	434	87	0.67 [0.35 – 1.29]
Centre-Ouest	390	121	0.94 [0.45 – 1.99]
Centre-Sud	217	51	0.66 [0.28 – 1.54]
Est	376	63	0.40 [0.22 – 0.74]**
Haut-Bassin	509	89	0.39 [0.24 – 0.66]***
Nord	420	72	0.66 [0.28 – 1.58]
Plateau Central	236	62	1.01 [0.37 – 2.75]
Sahel	315	4	0.02 [0.01 – 0.15]***
Sud-Ouest	214	148	5.63 [1.98 – 16.01]***

Model is adjusted for age, sex, living environment, education, marital status, occupational status and tobacco consumption. Alcohol consumption includes the different levels of consumption

* p = 0.05, ** p = 0.01, *** p < 0.001

For peer review only - http://bmjopen.bmj.com/site/about/guidelines.xhtml

For peer review only

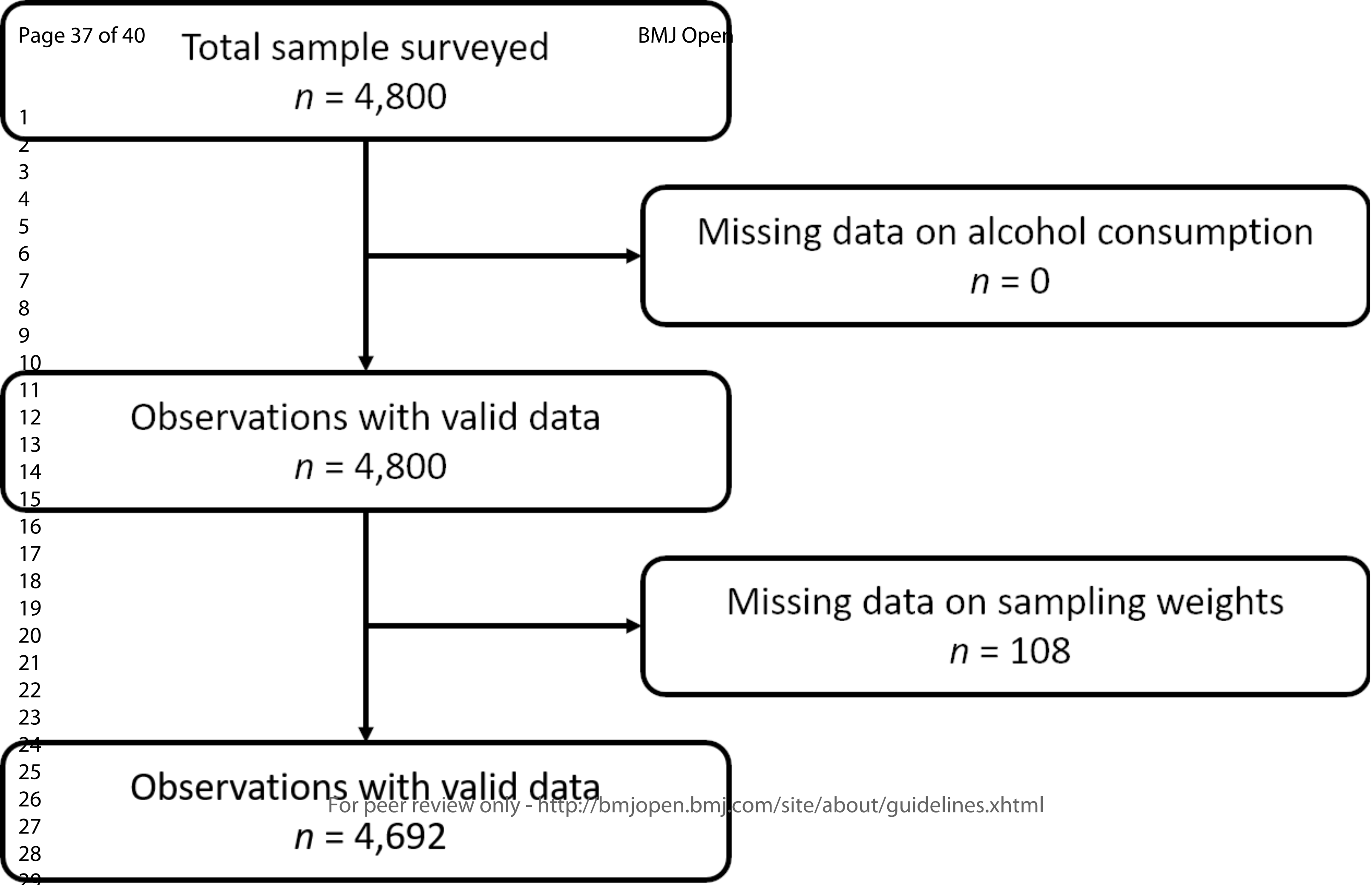

For peer review only - http://bmjopen.bmj.com/site/about/guidelines.xhtml

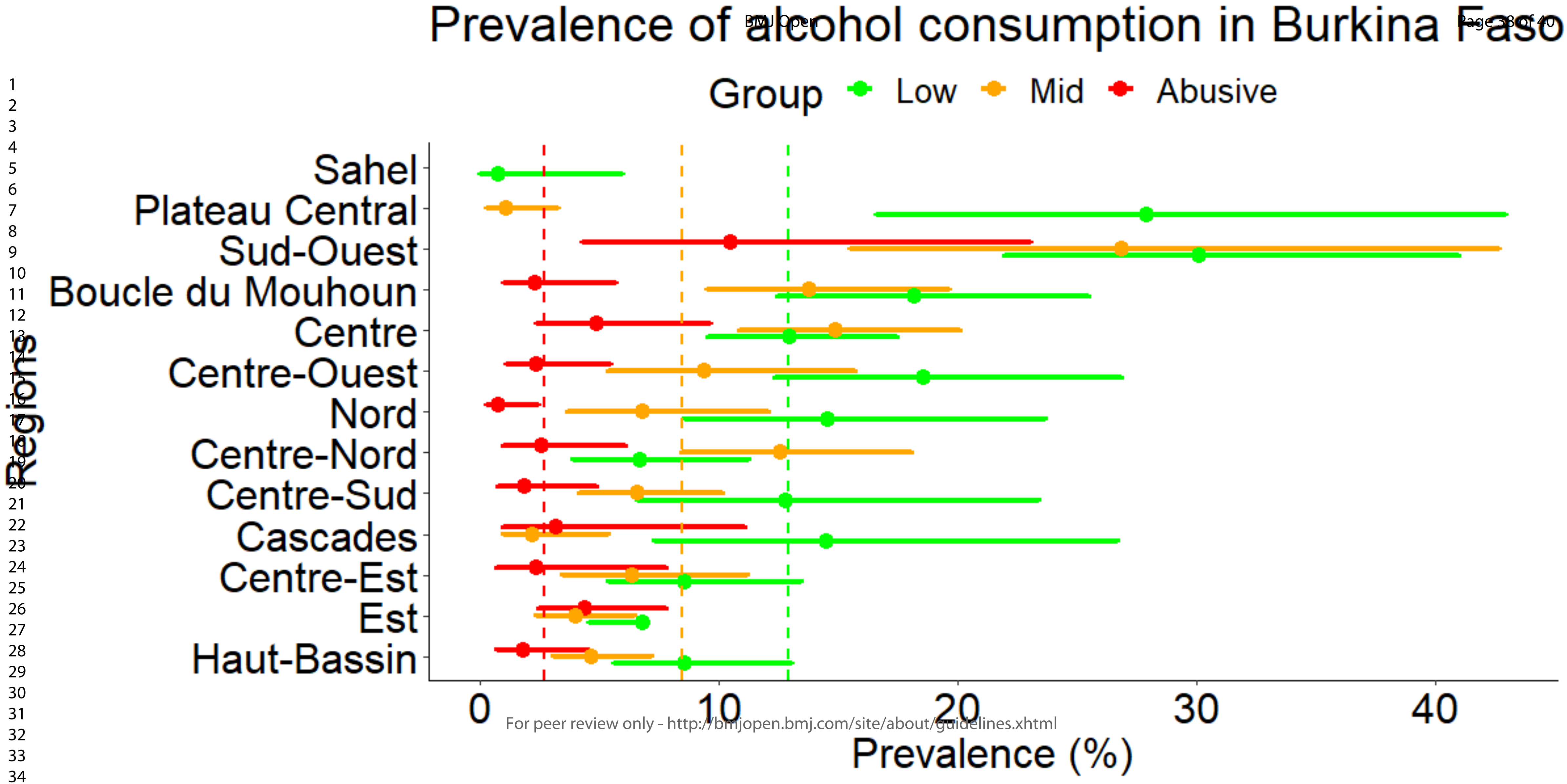

For peer review only - http://bmjopen.bmj.com/site/about/guidelines.xhtml

STROBE Statement—Checklist of items that should be included in reports of ***cross-sectional studies***

	Item No	Recommendation	Page
**Title and abstract**	1	(*a*) Indicate the study's design with a commonly used term in the title or the abstract	Yes
		(*b*) Provide in the abstract an informative and balanced summary of what was done and what was found	Yes
**Introduction**			
Background/rationale	2	Explain the scientific background and rationale for the investigation being reported	p.3
Objectives	3	State specific objectives, including any prespecified hypotheses	p.4
**Methods**			
Study design	4	Present key elements of study design early in the paper	Data source on p.5-6, sampling p.6
Setting	5	Describe the setting, locations, and relevant dates, including periods of recruitment, exposure, follow-up, and data collection	p.6
Participants	6	(*a*) Give the eligibility criteria, and the sources and methods of selection of participants	STEPs survey defined by WHO (p.6)
Variables	7	Clearly define all outcomes, exposures, predictors, potential confounders, and effect modifiers. Give diagnostic criteria, if applicable	p.7 and Table 1 and 2
Data sources/ measurement	8*	For each variable of interest, give sources of data and details of methods of assessment (measurement). Describe comparability of assessment methods if there is more than one group	NA
Bias	9	Describe any efforts to address potential sources of bias	NA
Study size	10	Explain how the study size was arrived at	p.5-6
Quantitative variables	11	Explain how quantitative variables were handled in the analyses. If applicable, describe which groupings were chosen and why	Definition of recoded exposure variables is presented in Table 1
Statistical methods	12	(*a*) Describe all statistical methods, including those used to control for confounding	p.7-8
		(*b*) Describe any methods used to examine subgroups and interactions	Multivariable logistic regression models and adjusted OR have been used (p.8 and in the results section)
		(*c*) Explain how missing data were addressed	Flow of the study participants is presented in Figure 1
		(*d*) If applicable, describe analytical methods taking account of sampling strategy	All data were weighted according to the cluster sampling design of the survey using strata and primary

For peer review only - http://bmjopen.bmj.com/site/about/guidelines.xhtml

			sampling units to allow the sample to be nationally representative (p.6)
		(*e*) Describe any sensitivity analyses	NA
**Results**			
Participants	13*	(a) Report numbers of individuals at each stage of study—eg numbers potentially eligible, examined for eligibility, confirmed eligible, included in the study, completing follow-up, and analysed	Figure 1
		(b) Give reasons for non-participation at each stage	NA
		(c) Consider use of a flow diagram	Figure 1
Descriptive data	14*	(a) Give characteristics of study participants (eg demographic, clinical, social) and information on exposures and potential confounders	Table 2
		(b) Indicate number of participants with missing data for each variable of interest	Figure 1
Outcome data	15*	Report numbers of outcome events or summary measures	Table 3
Main results	16	(*a*) Give unadjusted estimates and, if applicable, confounder-adjusted estimates and their precision (eg, 95% confidence interval). Make clear which confounders were adjusted for and why they were included	Table 3
		(*b*) Report category boundaries when continuous variables were categorized	Table 1
		(*c*) If relevant, consider translating estimates of relative risk into absolute risk for a meaningful time period	NA
Other analyses	17	Report other analyses done—eg analyses of subgroups and interactions, and sensitivity analyses	Multivaiate analysis are presented in Table 3 and explained in the method p.6-8
**Discussion**			
Key results	18	Summarise key results with reference to study objectives	First paragraphs of the discussion (p.11)
Limitations	19	Discuss limitations of the study, taking into account sources of potential bias or imprecision. Discuss both direction and magnitude of any potential bias	Start of the discussion (p.11)
Interpretation	20	Give a cautious overall interpretation of results considering objectives, limitations, multiplicity of analyses, results from similar studies, and other relevant evidence	p.11-12
Generalisability	21	Discuss the generalisability (external validity) of the study results	p.12-13
**Other information**			

For peer review only

Funding	22	Give the source of funding and the role of the funders for the present study and, if applicable, for the original study on which the present article is based	None

*Give information separately for exposed and unexposed groups.

Note: An Explanation and Elaboration article discusses each checklist item and gives methodological background and published examples of transparent reporting. The STROBE checklist is best used in conjunction with this article (freely available on the Web sites of PLoS Medicine at http://www.plosmedicine.org/, Annals of Internal Medicine at http://www.annals.org/, and Epidemiology at http://www.epidem.com/). Information on the STROBE Initiative is available at www.strobe-statement.org.

For peer review only

Alcohol consumption and associated risk factors in Burkina Faso: results of a population-based cross-sectional survey.

Journal:	*BMJ Open*
Manuscript ID	bmjopen-2021-058005.R1
Article Type:	Original research
Date Submitted by the Author:	06-Dec-2021
Complete List of Authors:	Bonnechère, Bruno; Hasselt University, REVAL Rehabilitation Research Center<br>Samadoulougou, Sékou; Quebec Heart and Lung Institute Research Center; Laval University<br>Cisse, Kadari; Institut de Recherche en Sciences de la Sante, Biomedical et santé publique; Université Libre de Bruxelles- Ecole de santé publique, Centre de Recherche en Epidémiologie, Biostatistiques et Recherche Clinique<br>Tassembedo, Souleymane; Centre MURAZ, Programme de Recherche sur les maladies Infectieuses<br>Kouanda, Seni; Institut de Recherche en Sciences de la Sante<br>Kirakoya-Samadoulougou, Fati; Université Libre de Bruxelles, Biostatistics, and Clinical Research
**Primary Subject Heading**:	Epidemiology
Secondary Subject Heading:	Addiction, Global health, Health policy, Public health
Keywords:	EPIDEMIOLOGY, PUBLIC HEALTH, TOXICOLOGY

SCHOLARONE™
Manuscripts

I, the Submitting Author has the right to grant and does grant on behalf of all authors of the Work (as defined in the below author licence), an exclusive licence and/or a non-exclusive licence for contributions from authors who are: i) UK Crown employees; ii) where BMJ has agreed a CC-BY licence shall apply, and/or iii) in accordance with the terms applicable for US Federal Government officers or employees acting as part of their official duties; on a worldwide, perpetual, irrevocable, royalty-free basis to BMJ Publishing Group Ltd ("BMJ") its licensees and where the relevant Journal is co-owned by BMJ to the co-owners of the Journal, to publish the Work in this journal and any other BMJ products and to exploit all rights, as set out in our licence.

The Submitting Author accepts and understands that any supply made under these terms is made by BMJ to the Submitting Author unless you are acting as an employee on behalf of your employer or a postgraduate student of an affiliated institution which is paying any applicable article publishing charge ("APC") for Open Access articles. Where the Submitting Author wishes to make the Work available on an Open Access basis (and intends to pay the relevant APC), the terms of reuse of such Open Access shall be governed by a Creative Commons licence – details of these licences and which Creative Commons licence will apply to this Work are set out in our licence referred to above.

Other than as permitted in any relevant BMJ Author's Self Archiving Policies, I confirm this Work has not been accepted for publication elsewhere, is not being considered for publication elsewhere and does not duplicate material already published. I confirm all authors consent to publication of this Work and authorise the granting of this licence.

For peer review only

Alcohol consumption and associated risk factors in Burkina Faso: results of a population-based cross-sectional survey.

Bonnechère Bruno[1*], Samadoulougou Sékou[2,3], Cissé Kadari[4,5],Tassembedo Souleymane[6], Kouanda Seni[5,7], Kirakoya-Samadoulougou Fati[4]

[1] REVAL Rehabilitation Research Center, Faculty of Rehabilitation Sciences, Hasselt University, Diepenbeek, Belgium

[2] Evaluation Platform on Obesity Prevention, Quebec Heart and Lung Institute Research Center, Quebec City, QC G1V 4G5, Canada

[3] Centre for Research on Planning and Development (CRAD), Laval University, Quebec, G1V 0A6, Canada

[4] Centre de Recherche en Epidémiologie, Biostatistiques et Recherche Clinique, Ecole de Santé Publique, Université libre de Bruxelles, Brussels, Belgium

[5] Institut de recherche en sciences de la santé (IRSS), Ouagadougou, 03 BP 7102, Burkina Faso

[6] Département de recherche clinique, centre Muraz, Bobo-Dioulasso, Burkina Faso

[7] Institut Africain de Santé publique (IASP), Ouagadougou, 12 BP 199, Burkina Faso

* corresponding author : bruno.bonnechere@uhasselt.be

Prof. Dr. Bonnechère Bruno

Agoralaan Gebouw A

3590 Diepenbeek

Belgium

For peer review only

For peer review only - http://bmjopen.bmj.com/site/about/guidelines.xhtml

Word count: 3681

Tables: 5

Figures: 2

For peer review only - http://bmjopen.bmj.com/site/about/guidelines.xhtml

Abstract

Objectives: Lifestyle modifiable risk factors are a leading preventable cause of non-communicable diseases (NCDs). Amongst them, alcohol consumption are the most important. Studies characterizing the prevalence of alcohol consumption in low-income countries are lacking. This study describes the prevalence of different levels of alcohol consumptions in Burkina Faso and its associated factors.

Design: Data from the 2013 Burkina Faso World Health Organization (WHO) Stepwise approach to Surveillance (STEPS), were analyzed. The prevalence of alcohol consumption over the last 30 days was recorded into categories according to WHO recommendations: low, mid, or heavy alcohol consumption. Multinomial logistic regression analyses identified factors associated with the different levels of alcohol consumption use.

Setting: Population-based cross-sectional survey

Participants: Overall, 4692 participants of both sexes aged 25 to 64 years were included in the study.

Results: In the whole sample 3,559 participants (75.8% [72.5 – 78.7]) were not consuming any alcohol, 614 (12.9% [10.9 – 15.3]) had a low alcohol consumption, 399 (8.5% [7.1 – 10.1]) a mid alcohol consumption and 120 (2.7% [2.0 – 3.7]) a heavy consumption. Age was associated with alcohol intake with a gradient effect, older people having a higher level of consumptions (AOR: 2.36, 95%CI [1.59 – 3.51] for low consumption, 2.50 [1.54 – 4.07] for mid consumption and 2.37 [1.01 – 5.92] for abusive consumption in comparison with no consumption). Tobacco consumption was also significantly associated with alcohol intake with a gradient effect, those

For peer review only - http://bmjopen.bmj.com/site/about/guidelines.xhtml

with higher tobacco consumption being at higher risk of abusive alcohol intake (AOR = 6.08 [2.75 – 13.4] for moderate consumption and 6.58 [1.96 – 22.11] abusive consumption).

Conclusion: Our data showed an important burden of alcohol consumption in Burkina Faso with age and tobacco effect. To effectively reduce alcohol consumption in Burkina Faso, comprehensive control and prevention campaigns should consider these associated factors.

Keywords

Alcohol consumption; Abusive alcohol consumption; Burkina Faso; Risk factors; Surveillance; WHO STEPS survey

For peer review only

For peer review only - http://bmjopen.bmj.com/site/about/guidelines.xhtml

Article summary

Strengths and Limitations of this study

- To the best of our knowledge, this study is the first national representative study on alcohol consumption within the adult population of Burkina Faso.
- This study presents alcohol consumption level and associated risk factors (age and tobacco consumption) in Burkina Faso using a large representative cohort of 4672 participants.
- The main limitations of this study are that we used results from 2013 (latest results available at large scale) and that the prevalence may have change due to the recent implementation of alcohol policies to limits the consumption.
- Another limitation is that alcohol consumptions and its associated risk factors were obtained during interviews (memory and social desirability biases).

For peer review only

For peer review only - http://bmjopen.bmj.com/site/about/guidelines.xhtml

Background

The increase in life expectancy [1] combined with unhealthy behaviors and physical inactivity [2] are linked to a rise in non-communicable diseases (NCDs) such as cardiovascular disease, cancer, chronic respiratory disease, and diabetes [3]. Currently, the leading causes of ill health in the world, NCDs account for seven out of ten deaths worldwide [4].

Low and mid-income countries are facing the emergence of non-communicable diseases (NCDs) [5]. Although some risk factors have been identified such as high blood pressure [6], lack of physical activity [7], inadequate diet [8], etc., there is still a lack of comprehensive data on other modifiable risk factors, in particular tobacco and alcohol consumption [9], in Burkina Faso.

Recently, clear evidence of increased alcohol consumption and attributable harm in many low and mid-income countries has been highlighted [10]. Furthermore, it is predicted to have a more harmful effect if an effective policy is not adopted [11]. Currently, the prevalence of alcohol intake among the African adult population is about 30% [12], which is lower compared to the rest of the world where 40% of the world's adult population consumes alcohol and the average consumption per drinker is 17.1 liters per year. Interestingly, the prevalence of lifetime abstention, the level of alcohol consumption, and the drinking patterns vary widely across regions of the world [13]. For example, Eastern Europe and Southern Africa had the most detrimental pattern of drinking scores. At the same time, Europe (Eastern and Central) and sub-Saharan Africa (Southern and West) are the most important consumers of alcohol [13]. In Africa, among current drinkers, the prevalence of heavy drinking varied between 7% and 77%, and the prevalence of daily light drinkers varied between 0% and 21%. Overall,

For peer review only

For peer review only - http://bmjopen.bmj.com/site/about/guidelines.xhtml

drinking patterns varied significantly between and within the examined African countries [14], more recent studies showed the same trends in South Africa and Ethiopia [15,16].

One of the major current concerns related to alcohol consumption in Africa is that alcohol companies have targeted Africa as a new market. With the expected increases in the number of potential new alcohol consumers, especially young people, and women, the African continent has indeed been identified by the alcohol beverage industry and market researchers as a key area for alcohol market growth [17,18]. It is, therefore, of particular importance to identify subjects the most at risk of alcohol consumption. Currently, two major risk factors have been identified worldwide: gender with the male being more at risk [19] and the socioeconomic level where the effect is less clear as the associations between socioeconomic disadvantage and heavier drinking vary depending on country-level income [20]. Pregnant mothers are particularly more vulnerable due to the fetus's susceptibility [21–24].

Another important aspect highlighting the need for local studies is that it has been shown that there are significant disparities in alcohol use between Africans (Ghanaians) residing in Europe and Africa, indicating that migration has a significant impact on drinking habits and also implying that alcohol reduction initiatives may need distinct techniques.[25]

There is currently a lack of information about the prevalence of alcohol consumption in Burkina Faso and the associated risk factors. Differences in cultural factors (e.g., beliefs and practices) may influence health status, but social, economic, and structural determinants of health during people's lifespans appear to be associated with health inequities between ethnic groups as well. Therefore, cultural influences should not be overemphasized as discrete explanatory factors for health inequities [26].

For peer review only - http://bmjopen.bmj.com/site/about/guidelines.xhtml

A first national survey was conducted in 2013 using the WHO STEPwise approach to risk factors surveillance (STEPS). The STEPS survey is a simple, standardized method for collecting, analyzing, and disseminating data in WHO member countries. It covered a representative sample of the adult population. The first analyses performed were about the evaluation of cardiovascular risk [27]. In a previous study, we show that tobacco consumption was highly correlated with alcohol consumption in men [28]. Therefore, this study aims to study the prevalence of alcohol consumption and its associated risk factors. Such kind of analysis is needed to drive more efficient prevention campaigns for both alcohol and tobacco consumption.

Methods

Study settings

Burkina Faso is a landlocked country in West Africa of 272 967.47 km^2. The country is divided into 13 administrative regions and is limited in the North and West by Mali, in the East by Niger, and in the South by Benin, Togo, Ghana, and Côte d'Ivoire.

In 2020, the population was estimated at 21,510,181 inhabitants [29]. The majority of the population (77.30%) lives in rural areas and relies on agriculture and livestock as the main source of income [30]. The median age of the population is 17.9 years old. In 2018, the total fertility rate was 5.2 and the life expectancy at birth was 62.3 years in 2019 [31]. Like most low-income countries, Burkina Faso must face the double burden of infectious and chronic diseases. The country is regularly confronted with outbreaks such as measles, cerebrospinal

For peer review only - http://bmjopen.bmj.com/site/about/guidelines.xhtml

meningitis and, malaria [32], while NCDs constitute a rising public health problem with only limited financial resources allocated.

In Burkina Faso, several sources of alcohol manufacturing exist modern brewery and legally imported alcohol, prohibited alcohol mostly imported from neighboring countries and traditional beer manufacturers. Burkina Faso is a secular country with more than half of the population declared as being of Islamic faith (61.6%), 22.5% Christians, and 15.4% traditional religions [33].

Harmful alcohol use and its adverse events (i.e., road traffic accidents, domestic violence) are however important and raising public health concerns in Burkina Faso [34].

Study design

This is an analytical cross-sectional study of the WHO STEPS survey conducted in Burkina Faso in 2013. Complete details on the methodology and sampling procedure were described in the WHO STEPS 2013 Burkina report [35], and in previous studies using this database [27,28,36]. The participants were selected using a three-stage cluster sampling process described in the sampling method.

Study population

Participants of the study were adults of both sexes aged 25 to 64 years old who had been living in Burkina Faso for at least six months. Exclusion criteria were: people with disabilities hampering their ability to answer the questions (e.g., intellectual disabilities, serious mental disorders, cognitive impairment, etc.).

For peer review only - http://bmjopen.bmj.com/site/about/guidelines.xhtml

Patient and public involvement

No patients were involved in the study design or analysis of the anonymous data set.

Sampling

The STEPS survey of Burkina Faso was conducted on a representative sample of 4,800 individuals, the response rate of the STEPS survey was 99.1% in Burkina Faso [32]. After excluding participants with missing information about sampling weight, 4,692 individuals were included in the present analysis. The diagram flow of study participants is presented in Figure 1. A three levels sampling frame was performed. In the first stage, geographic areas were stratified into rural and urban, and EAs were selected with probability proportional to their size from both strata. A total of 240 EAs were selected: 185 from rural areas and 55 from urban areas. In the second stage, 20 households were selected from each EA. In the third stage, one person aged from 25 to 64 years in each household was selected using the Kish method.

Data collection

A full description of the study design and the data collection has been published elsewhere [27,36]. Briefly, all data on alcohol consumption were collected using a standardized questionnaire during face-to-face interviews in the language most spoken by the respondent (Step 1 of the STEP survey). The questionnaires were pre-tested on the field before being used for the national survey. Alcohol consumption was assessed with the question '*Have you ever consumed any alcohol such as beer, wine, spirits or dolo*'? If the answer is yes, the current alcohol consumption was assessed with the questions '*During the past 30 days, how many days did you consume alcohol drinks?' and 'When you are drinking, what is the average number of drinks* ?'. Four levels were defined: no consumption, low, mid, and abusive

For peer review only - http://bmjopen.bmj.com/site/about/guidelines.xhtml

For peer review only

consumption (see Table 1 for WHO definition). According to the WHO recommendation, the level defining the different levels of consumption have been defined differently for males and females.

Other information such as demographic information, anthropometric and behavioral measurements were also collected (Step 2). In our study, all the variables used in the manuscript were collected in Step I.

Study variables

The dependent variable was the mean alcohol consumption in the last 30 days. First, we analyzed alcohol consumption to define the profile of the drinker.

The independent variables were: age, sex, education, marital status, occupational status, and tobacco consumption (see Table 1 for the definition of the variables).

Data analysis and processing

Some independent quantitative variables were transformed into categorical variables. The list of the variables with the recoded variables is presented in Table 1. Categorical variables were described using proportions. The Chi-squared test was used in univariable analysis to test the association between the outcome and the categorical variables. Multivariable analysis was then performed: we used a logistic regression model fitted using stepwise backward regression modeling. We first analyzed the profile and levels of alcohol consumption using multinomial logistic regression defining no alcohol consumption as the reference value. Then we identify the profile of the abusive consumer, following WHO recommendations [37], amongst current drinkers using logistic regression grouping low and mid consumption as reference group.

For peer review only - http://bmjopen.bmj.com/site/about/guidelines.xhtml

Adjusted Odds Ratios (AORs) were calculated for the studied variables and presented with 95% confidence intervals. We then analyzed discrepancies between the different regions using adjusted AORs for the different regions.

All the analyses were carried out using the survey function with the sampling weight option to account for the sampling design.

Statistics have been conducted in STATA (version 13) and RStudio (version 1.1.442) with R version 3.4.4).

Results

Characteristics of the population are presented in Table 2. Most of the participants were rural residents (79.7%). The population was predominantly young, with the 25-34 years group representing 41.9%. Women were more represented, with 54.3% of the total sample.

In the whole population 3,559 (75.8% [72.5 – 78.7]) were not consuming any alcohol, 614 (12.9% [10.9 – 15.3]) had a low alcohol consumption, 399 (8.5% [7.1 – 10.1]) a mid alcohol consumption and 120 (2.7% [2.0 – 3.7]) an abusive consumption. There are important differences between the different regions of the countries; the results are presented in Figure 2. In the *Sahel* region, the prevalence is null while in the Sud-Ouest regions the prevalence for all the levels is statistically significantly higher than the country's average values.

The prevalence of abusive consumption was not different in urban setting and rural setting (4.3 % [2.5 – 7.2] versus 2.1% [1.5 – 3.1], p = .19). The repartition of consumption was statistically significantly different between urban and rural environment (*p* =.001): There was a higher proportion of low alcohol consumption is the rural area (13.1% compared to 12.7%)

For peer review only - http://bmjopen.bmj.com/site/about/guidelines.xhtml

while in the urban area there is more mid (10.0% Vs. 8.0%) and high consumption (4.3% Vs. 2.2%).

In multivariable analysis (Table 3), first concerning the alcohol consumption, there is a statistically significant gradient effect of the age regardless of the level of consumption. We found that compared to no alcohol consumption, the odds of engaging in low alcohol consumption was 1.46 times higher among people with 35-44 years compared to those with 25-34 years old (AOR = 1.46 [1.09 – 1.95]). The odds was 1.76 times higher (AOR: 1.76 [1.34 – 2.40]) and 2.36 [1.59 – 3.51] for the 45-54 and 55-64 years old respectively compared to the 25-34 years old group. Regarding mid consumption this odds was 1.55 [1.14 – 2.12], 1.81 [1.25 – 2.62], and 2.50 [1.54 – 4.07] times higher for 35-44 years, 45-54 years and 55-64 years respectively compared to 25-34 years old. A similar results were found when considering abusive alcohol consumption (AOR = 1.86 [1.06 – 3.27], 2.67 [1.36 – 5.25], 2.37 [1.01 – 5.92] for abusive consumption. Interestingly, the effect of age is different regarding the level of consumption: there is an increased risk in male for low (AOR = 2.68 [1.96 – 3.67]) and abusive consumption (AOR = 2.89 [1.56 – 2.37]), while there is a protective effect against moderate consumption (AOR = 0.38 [0.26 – 0.55]). The level of education is also associated with an increased risk, regardless of consumption level (AOR = 2.08 [1.07 – 4.03], 2.97 [1.48 – 5.92], and 3.50 [1.34 – 9.15] for low, mid, and abusive consumption respectively for participants who reached secondary levels in comparison of people without any education).

There is also an important gradient effect associated with tobacco consumption: the consumption of small tobacco quantity (<5 cigs/day) is associated with an increased risk of low alcohol consumption (AOR = 1.72 [1.13 – 2.62]) while the consumption of high quantity of

For peer review only - http://bmjopen.bmj.com/site/about/guidelines.xhtml

tobacco is associated with mid and abusive alcohol consumption (AOR = 6.08 [2.75 – 13.4] and 6.58 [1.96 – 22.11] respectively).

Finally, we computed the risk of having abusive consumption in those consuming alcohol (Table 4). Sex is an important risk factor with increased risk for the men compared to women (AOR = 2.53 [1.38 – 4.68]) as well as the occupational status with jobless people and housemaker associated with a decreased risk of having abusive consumption (AOR = 0.05 [0.01 – 0.47] and AOR = 0.14 [0.02 – 0.78] respectively).

The risk also varied from the different regions, AORs for the different regions of the countries are presented in Table 5. We observed that the *Sud-Ouest* region has a higher risk of consumption compared to the rest of the country. In the *Sahel*, *Est,* and *Haut-Bassin*, the risks of consumption are significantly lower.

Discussion

We report here the results of the first nationally representative survey on the prevalence and risk factors for alcohol consumption in Burkina Faso.

The overall prevalence of people with abusive alcohol consumption is 2.7% [2.0 – 3.7]. This consumption is lower compared to other African countries [12] and the rest of the world [13]. However, this prevalence might be underestimated because even though Burkina Faso is a secular country, the majority of its inhabitants have a religious faith that prohibits alcohol consumption that could prevent some people from declaring their alcohol intake, this might be particularly true in the *Sahel* region. Also, alcohol consumption is blamed by society and could also lead to an under-declaration (positive social perception bias) [38].

For peer review only - http://bmjopen.bmj.com/site/about/guidelines.xhtml

We also found differences in the different regions of the country with the highest levels of consumption found in the *Sud-Ouest* region. In this region, almost every household produces local beer (*'dolo'*) this might explain this observation. The income disparities and the alcohol availability between the different regions could also explain those differences [39]. From a public health perspective, this region also has the highest prevalence of Hepatitis B and C [40]. The inhabitants of these regions seem to cumulate the hepatotoxic risk of Hepatitis and alcohol consumption. We found a null prevalence in the Sahel region, this is an entirely Muslim region and the desirability bias may be more important compared to other regions.

We also found gender differences with an increased risk in men compared to women for low and abusive alcohol consumption but an increased risk of moderate consumption for women. It is important to specify here that, following the WHO guidelines, the thresholds used to define the different levels of alcohol consumption are different for men and women (33 to 50% lower for women compared to men). The risk of abusive consumption in drinkers is more important in men compared to women.

In most countries, the prevalence is higher in men compared to women [19,41], but when adjusting for multiple factors such as social supports and financial aspects, this effect seems less important. Compared to men, more women are lifetime abstainers, drink less, and are less likely to engage in problem drinking. Of note is that women drinking excessively develop more severe medical problems than men. Biological (sex-related) factors, including differences in alcohol pharmacokinetics as well as its effect on brain function and the levels of sex hormones, may contribute to some of those differences [42]. Since pregnant women are particularly vulnerable, we performed a sub-group analysis. A subgroup of 299 out of the 2,449 (12.2%) women was pregnant during the survey, amongst them, 6 (3.9%) reported

For peer review only - http://bmjopen.bmj.com/site/about/guidelines.xhtml

alcohol consumption, which is statistically lower compared to non-pregnant women (12.6%, $p = .001$).

Interestingly we observed that age was significantly associated with alcohol consumption with a gradient effect of age on alcohol consumption, but it seems that the level of consumption is not influenced by gender as the AOR are relatively similar for the different categories. The influence of age on alcohol consumption is still unclear and not well documented in the literature, except for binge drinking, where young adults are the most at risk [43]. However, this result should be interpreted carefully, considering our study design. This could be the results of preventive campaigns as reflected by a cohort effect A large Australian study including 7 cross-sectional waves showed indeed that male cohorts born between 1965 and 1974 and female cohorts born between 1955 and 1974 reported higher rates of drinking participation (P < 0.05), while the most recent cohorts (born in the 1990s) had lower rates of participation (P < 0.01) [44].

Concerning the risk of switching to an abusive consumption among drinkers only two factors have been identified: the risk is increased in males (as for the general consumption) and decreased with the occupational status, probably due to financial constraints.

The association with tobacco consumption is probably one of the most important from a public health view because of the comorbidities and the double burden it presents for the population. In a previous study, we identified people most at risk of tobacco consumption in Burkina Faso: tobacco smoking among men was significantly associated with increased age and alcohol consumption. Analysis of risk factors for other tobacco use stratified by gender shows that age, education, residence, and alcohol consumption were significantly associated with consumption for women, age, and alcohol consumption for men [28].

For peer review only - http://bmjopen.bmj.com/site/about/guidelines.xhtml

For peer review only

As recommended by the WHO, in this paper we presented risk factors associated with abusive alcohol consumption because of the proven negative effects. It is, however, interesting to note that from a medical point of view a limited and reasonable alcohol consumption could have some health-related benefits: for example, the relation between dementia and cognitive disorders is not linear and limited alcohol consumption has a protective role for dementia [45–47] or the protective effect of alcohol on cardiovascular risk, previously known as *'the French paradox'* [48–50]. However, these results must be interpreted with caution, especially since it is known that even in low doses, alcohol consumption transiently increases the risk of cardiovascular accidents [51]. Another important point is that considering the prevention side experiences showed that adopting a too strong position by prohibiting any consumption or behavior will lead to poor results [52,53]. Considering these two aspects low alcohol consumption could be considered as acceptable. Alcohol policies that regulate the physical availability of alcohol are associated with lower alcohol consumption in low- and middle-income countries [54]. Burkina Faso is at an embryonic stage in this area. Indeed, the country just had its first specifications plan on alcoholic beverages production, importation, and selling adopted in 2020 [55]. This plan was jointly approved by the Ministry of Trade and the companies involved in such activities. Some additional measurements have been adopted by the government. These include the prohibition of advertisements for tobacco and alcohol products and, since September 19, 2019, the government also banned the production, importation, and marketing of liqueurs and other spirit drinks in plastic bags and bottles of less than 30 centiliters to limit the availability [56]. This plan needs to be rigorously implemented first and then rapidly incremented to a stronger legislative law to positively impact behavior and health outcomes.

For peer review only - http://bmjopen.bmj.com/site/about/guidelines.xhtml

The main limitation of this study is that alcohol (and tobacco) consumptions were obtained during interviews and is therefore dependent on the faith of the participants. There is, therefore, both a risk of memory bias and social desirability, probably more marked during pregnancy. It can thus be estimated that the numbers and prevalence obtained in this survey underestimate the actual consumption. Another potential limitation is that some well-known risk factors for alcohol consumption were not included in the study because data on these variables have not been collected during the STEPS survey. Part of such variables is socioeconomic status. It is a transversal study; therefore there is a risk of survival bias indicating that older participants with high consumption may die prematurely due to this consumption [57]. The last limitation is that we used data collected in 2013, these data may not represent the actual situation anymore. We have indeed seen that the government has taken measurements to restrict and limit alcohol consumption, therefore the prevalence may be more important than the current situation. However, in this paper, we mainly focus on the risk factor and we do not think that the risk factor has been much modified between 2013 and today. Another STEP survey was planned for 2020 to have more recent numbers but unfortunately, the survey could not take place due to the COVID-19 pandemic. In the future the data of this survey could be used to monitor the level of consumption and determine if these measurements are working and if other measures must be taken.

Despite these limitations, given the study design (cluster sampling design) and the sample size, the results of this study can be extended to the whole of Burkina Faso.

For peer review only - http://bmjopen.bmj.com/site/about/guidelines.xhtml

Conclusion

In this study, we reported on the burden of alcohol consumption and associated risk factors in a nationally representative sample of adults in Burkina Faso. Our data showed an important burden of alcohol consumption in Burkina Faso. Tobacco consumption is an important modifiable risk factor associated with alcohol consumption. Zone-specific interventions are needed given the higher burden in urban centers and some specific regions such as the *Sud-Ouest* region. Health policies in Burkina Faso must henceforth account for the control of alcohol and tobacco consumption since there is a strong relationship between those two important risk factors of NCDs. Alcohol policies that regulate the physical availability of alcohol are associated with lower alcohol consumption and should, therefore, be implemented in Burkina Faso to reduce the burden of alcohol consumption.

List of abbreviation

DHS: Demographic and Health Surveys

NCD: Non-communicable disease

SSA: sub-Saharan Africa

STEPS: WHO STEPwise approach to surveillance

WHO: World Health Organization

For peer review only - http://bmjopen.bmj.com/site/about/guidelines.xhtml

Declarations

Ethics approval and consent to participate Consent for publication

The protocol of the STEPS survey was reviewed and approved by the Ethics Committee for Health Research of the Ministry of Health of Burkina Faso, which gave clearance in accordance with regulations in force (Deliberation No. 2012–12- 092 of 05 December 2012). Written informed consent was systematically sought and obtained from all participants before inclusion in the study. The confidentiality of study participants was fully respected and the analyses performed did not identify any participant. For this study, we obtained authorization from the General Directorate of Health to reanalyze the data, and the confidentiality of study participants was preserved.

Availability of data and materials

The dataset of the STEPS survey that was used in this research is available at the Ministry of Health upon request. Other STEPS surveys can be obtained through the WHO (https://www.who.int/ncds/surveillance/steps/riskfactor/en/). Any request to reanalyze the data can be directed to Dr Brice Bicaba (bicababrico78@gmail.com). No further additional data are available.

Acknowledgments

The authors are thankful to all the people involved in the STEPS survey. Bruno Bonnechère thanks people who help him during the analysis of the data in Burkina Faso.

For peer review only - http://bmjopen.bmj.com/site/about/guidelines.xhtml

Competing interests

The authors declare that they have no competing interests.

Funding

Bonnechère Bruno received a travel grant from ARES (https://www.ares-ac.be/fr/) to conduct the data analysis in Burkina Faso (second analysis of the STEP survey), interpret the data with local epidemiologists and write the manuscript. Cissé Kadari is funded by ARES. The funding body had no role in study design, data collection and analysis, decision to publish, or preparation of the manuscript. Award/Grant number is not applicable

Author contributions

BB and FKS conceived the study. BB performed the analysis. BB, SS and CK proposed an early draft of the paper. FKS, SK, and ST made substantial contributions to the conception and design, analysis and interpretation of the data. KFS, SK and ST contributed significantly to revise the manuscript. All authors read and approved the final manuscript.

For peer review only

For peer review only - http://bmjopen.bmj.com/site/about/guidelines.xhtml

Figures captions

Figure 1: Consort Diagram describing study participants, exclusions and analytic sample size.

Figure 2 : Prevalence of the level of alcohol consumption in the different regions of Burkina Faso. Vertical lines indicate the mean prevalence for the whole country.

For peer review only

References

1 GBD 2015 DALYs and HALE Collaborators. Global, regional, and national disability-adjusted life-years (DALYs) for 315 diseases and injuries and healthy life expectancy (HALE), 1990-2015: a systematic analysis for the Global Burden of Disease Study 2015. *Lancet Lond Engl* 2016;**388**:1603–58. doi:10.1016/S0140-6736(16)31460-X

2 Lee I-M, Shiroma EJ, Lobelo F, *et al.* Effect of physical inactivity on major non-communicable diseases worldwide: an analysis of burden of disease and life expectancy. *Lancet Lond Engl* 2012;**380**:219–29. doi:10.1016/S0140-6736(12)61031-9

3 NCD Countdown 2030 collaborators. NCD Countdown 2030: worldwide trends in non-communicable disease mortality and progress towards Sustainable Development Goal target 3.4. *Lancet Lond Engl* 2018;**392**:1072–88. doi:10.1016/S0140-6736(18)31992-5

4 World Health Organization. World Health Statistics 2018: Monitoring health for the SDGs. 2018.

5 Jamison DT, Summers LH, Alleyne G, *et al.* Global health 2035: a world converging within a generation. *Lancet Lond Engl* 2013;**382**:1898–955. doi:10.1016/S0140-6736(13)62105-4

6 Kearney PM, Whelton M, Reynolds K, *et al.* Global burden of hypertension: analysis of worldwide data. *Lancet Lond Engl* 2005;**365**:217–23. doi:10.1016/S0140-6736(05)17741-1

7 Whiteford HA, Degenhardt L, Rehm J, *et al.* Global burden of disease attributable to mental and substance use disorders: findings from the Global Burden of Disease Study 2010. *Lancet Lond Engl* 2013;**382**:1575–86. doi:10.1016/S0140-6736(13)61611-6

8 Afshin A, Micha R, Webb M, *et al.* Effectiveness of Dietary Policies to Reduce Noncommunicable Diseases. In: Prabhakaran D, Anand S, Gaziano TA, *et al.*, eds. *Cardiovascular, Respiratory, and Related Disorders*. Washington (DC): : The International Bank for Reconstruction and Development / The World Bank 2017. http://www.ncbi.nlm.nih.gov/books/NBK525147/ (accessed 10 Sep 2019).

9 Juma PA, Mohamed SF, Matanje Mwagomba BL, *et al.* Non-communicable disease prevention policy process in five African countries. *BMC Public Health* 2018;**18**:961. doi:10.1186/s12889-018-5825-7

10 Shield K, Manthey J, Rylett M, *et al.* National, regional, and global burdens of disease from 2000 to 2016 attributable to alcohol use: a comparative risk assessment study. *Lancet Public Health* 2020;**5**:e51–61. doi:10.1016/S2468-2667(19)30231-2

11 Manthey J, Shield KD, Rylett M, *et al.* Global alcohol exposure between 1990 and 2017 and forecasts until 2030: a modelling study. *The Lancet* 2019;**393**:2493–502. doi:10.1016/S0140-6736(18)32744-2

For peer review only

12 World Health Organization. Global Status Report on Alcohol and Health—2014. 2014.

13 Shield KD, Rylett M, Gmel G, *et al.* Global alcohol exposure estimates by country, territory and region for 2005--a contribution to the Comparative Risk Assessment for the 2010 Global Burden of Disease Study. *Addict Abingdon Engl* 2013;**108**:912–22. doi:10.1111/add.12112

14 Clausen T, Rossow I, Naidoo N, *et al.* Diverse alcohol drinking patterns in 20 African countries. *Addict Abingdon Engl* 2009;**104**:1147–54. doi:10.1111/j.1360-0443.2009.02559.x

15 Probst C, Shuper PA, Rehm J. Coverage of alcohol consumption by national surveys in South Africa. *Addict Abingdon Engl* 2017;**112**:705–10. doi:10.1111/add.13692

16 Ayano G, Yohannis K, Abraha M, *et al.* The epidemiology of alcohol consumption in Ethiopia: a systematic review and meta-analysis. *Subst Abuse Treat Prev Policy* 2019;**14**:26. doi:10.1186/s13011-019-0214-5

17 Reuters. SABMiller Eyes Africa $2.5bn Investment. 2012.http://www.iol.co.za/business/international/sabmiller-eyes-africa-25bn-investment-1277086 (accessed 8 Jul 2019).

18 African Business. Alcohol Consumption Rising in Africa Despite Obstacles. https://africanbusinessmagazine.com/sectors/retail/alcohol-consumption-rising-in-africa-despite-obstacles/ (accessed 8 Jul 2019).

19 Chaiyasong S, Huckle T, Mackintosh A-M, *et al.* Drinking patterns vary by gender, age and country-level income: Cross-country analysis of the International Alcohol Control Study. *Drug Alcohol Rev* 2018;**37 Suppl 2**:S53–62. doi:10.1111/dar.12820

20 Huckle T, Romeo JS, Wall M, *et al.* Socio-economic disadvantage is associated with heavier drinking in high but not middle-income countries participating in the International Alcohol Control Study. *Drug Alcohol Rev* 2018;**37 Suppl 2**:S63–71. doi:10.1111/dar.12810

21 Martinez P, Røislien J, Naidoo N, *et al.* Alcohol abstinence and drinking among African women: data from the World Health Surveys. *BMC Public Health* 2011;**11**:160. doi:10.1186/1471-2458-11-160

22 Popova S, Lange S, Probst C, *et al.* Actual and predicted prevalence of alcohol consumption during pregnancy in the WHO African Region. *Trop Med Int Health TM IH* 2016;**21**:1209–39. doi:10.1111/tmi.12755

23 Sanou AS, Diallo AH, Holding P, *et al.* Maternal alcohol consumption during pregnancy and child's cognitive performance at 6-8 years of age in rural Burkina Faso: an observational study. *PeerJ* 2017;**5**:e3507. doi:10.7717/peerj.3507

24 Addila AE, Bisetegn TA, Gete YK, *et al.* Alcohol consumption and its associated factors among pregnant women in Sub-Saharan Africa: a systematic review and meta-analysis' as

For peer review only - http://bmjopen.bmj.com/site/about/guidelines.xhtml

given in the submission system. *Subst Abuse Treat Prev Policy* 2020;**15**:29. doi:10.1186/s13011-020-00269-3

25 Addo J, Cook S, Galbete C, *et al.* Differences in alcohol consumption and drinking patterns in Ghanaians in Europe and Africa: The RODAM Study. *PloS One* 2018;**13**:e0206286. doi:10.1371/journal.pone.0206286

26 Mosdøl A, Lidal IB, Straumann GH, *et al.* Targeted mass media interventions promoting healthy behaviours to reduce risk of non-communicable diseases in adult, ethnic minorities. *Cochrane Database Syst Rev* 2017;**2**:CD011683. doi:10.1002/14651858.CD011683.pub2

27 Soubeiga JK, Millogo T, Bicaba BW, *et al.* Prevalence and factors associated with hypertension in Burkina Faso: a countrywide cross-sectional study. *BMC Public Health* 2017;**17**:64. doi:10.1186/s12889-016-3926-8

28 Bonnechère B, Cissé K, Millogo Tiéba, *et al.* Tobacco use and associated risk factors in Burkina Faso: Results from a nationwide population-based survey. *BMC Public Health* 2019.

29 Ministère de l'économie et des finances. Projection démographique de 2007 à 2020 par région et province. Institut national de la statistique et de la démographie, Burkina Faso. http://www.cns.bf/IMG/pdf/projections_demographiques_sous_nationales.pdf

30 Institut National de la Statistique et de la Démographie (INSD); ICF International. Enquête Démographique et de Santé et à Indicateurs Multiples du Burkina Faso 2010. 2012.

31 Institut national de la statistique et de la démographie. BURKINA FASO MINISTERE DE L'ECONOMIE ET DES FINANCES: PROJECTIONS DEMOGRAPHIQUES DE 2007 A 2020 PAR REGION ET PROVINCE. http://www.cns.bf/IMG/pdf/projections_demographiques_sous_nationales.pdf

32 Ministère de la santé du Burkina Faso. Rapport de l'enquête nationale sur la prévalence des principaux facteurs de risque communs aux maladies non transmissibles (STEPS 2013). 2014.

33 La Croix A. Données géographiques et identité religieuse au Burkina Faso. Données Géographiques Identité Relig. Au Burkina Faso. https://africa.la-croix.com/statistiques/burkina-faso/ (accessed 28 Feb 2021).

34 Bonnet E, Lechat L, Ridde V. What interventions are required to reduce road traffic injuries in Africa? A scoping review of the literature. *PloS One* 2018;**13**:e0208195. doi:10.1371/journal.pone.0208195

35 WHO. STEPwise approach to chronic disease risk factor surveillance. https://www.who.int/ncds/surveillance/steps/burkina_faso/en/

For peer review only - http://bmjopen.bmj.com/site/about/guidelines.xhtml

36 Millogo T, Bicaba BW, Soubeiga JK, *et al.* Diabetes and abnormal glucose regulation in the adult population of Burkina Faso: prevalence and predictors. *BMC Public Health* 2018;**18**. doi:10.1186/s12889-018-5257-4

37 Kendagor A, Gathecha G, Ntakuka MW, *et al.* Prevalence and determinants of heavy episodic drinking among adults in Kenya: analysis of the STEPwise survey, 2015. *BMC Public Health* 2018;**18**:1216. doi:10.1186/s12889-018-6057-6

38 Jemmott JB, Ditto PH, Croyle RT. Judging health status: effects of perceived prevalence and personal relevance. *J Pers Soc Psychol* 1986;**50**:899–905. doi:10.1037//0022-3514.50.5.899

39 Dixon MA, Chartier KG. Alcohol Use Patterns Among Urban and Rural Residents: Demographic and Social Influences. *Alcohol Res Curr Rev* 2016;**38**:69–77.

40 Meda N, Tuaillon E, Kania D, *et al.* Hepatitis B and C virus seroprevalence, Burkina Faso: a cross-sectional study. *Bull World Health Organ* 2018;**96**:750–9. doi:10.2471/BLT.18.208603

41 Takahashi R, Wilunda C, Magutah K, *et al.* Correlates of alcohol consumption in rural western Kenya: A cross-sectional study. *BMC Psychiatry* 2017;**17**:175. doi:10.1186/s12888-017-1344-9

42 Erol A, Karpyak VM. Sex and gender-related differences in alcohol use and its consequences: Contemporary knowledge and future research considerations. *Drug Alcohol Depend* 2015;**156**:1–13. doi:10.1016/j.drugalcdep.2015.08.023

43 Kuntsche E, Kuntsche S, Thrul J, *et al.* Binge drinking: Health impact, prevalence, correlates and interventions. *Psychol Health* 2017;**32**:976–1017. doi:10.1080/08870446.2017.1325889

44 Livingston M, Raninen J, Slade T, *et al.* Understanding trends in Australian alcohol consumption-an age-period-cohort model. *Addict Abingdon Engl* 2016;**111**:1590–8. doi:10.1111/add.13396

45 Heffernan M, Mather KA, Xu J, *et al.* Alcohol Consumption and Incident Dementia: Evidence from the Sydney Memory and Ageing Study. *J Alzheimers Dis JAD* 2016;**52**:529–38. doi:10.3233/JAD-150537

46 Xu W, Wang H, Wan Y, *et al.* Alcohol consumption and dementia risk: a dose-response meta-analysis of prospective studies. *Eur J Epidemiol* 2017;**32**:31–42. doi:10.1007/s10654-017-0225-3

47 Gutwinski S, Schreiter S, Priller J, *et al.* Drink and Think: Impact of Alcohol on Cognitive Functions and Dementia - Evidence of Dose-Related Effects. *Pharmacopsychiatry* 2018;**51**:136–43. doi:10.1055/s-0043-118664

For peer review only

For peer review only - http://bmjopen.bmj.com/site/about/guidelines.xhtml

48 O'Keefe JH, Bhatti SK, Bajwa A, *et al.* Alcohol and cardiovascular health: the dose makes the poison…or the remedy. *Mayo Clin Proc* 2014;**89**:382–93. doi:10.1016/j.mayocp.2013.11.005

49 Fernández-Solà J. Cardiovascular risks and benefits of moderate and heavy alcohol consumption. *Nat Rev Cardiol* 2015;**12**:576–87. doi:10.1038/nrcardio.2015.91

50 Rehm J, Roerecke M. Cardiovascular effects of alcohol consumption. *Trends Cardiovasc Med* 2017;**27**:534–8. doi:10.1016/j.tcm.2017.06.002

51 Mostofsky E, Chahal HS, Mukamal KJ, *et al.* Alcohol and Immediate Risk of Cardiovascular Events: A Systematic Review and Dose-Response Meta-Analysis. *Circulation* 2016;**133**:979–87. doi:10.1161/CIRCULATIONAHA.115.019743

52 Cyril S, Smith BJ, Renzaho AMN. Systematic review of empowerment measures in health promotion. *Health Promot Int* 2016;**31**:809–26. doi:10.1093/heapro/dav059

53 Gelius P, Rütten A. Conceptualizing structural change in health promotion: why we still need to know more about theory. *Health Promot Int* 2018;**33**:657–64. doi:10.1093/heapro/dax006

54 Cook WK, Bond J, Greenfield TK. Are alcohol policies associated with alcohol consumption in low- and middle-income countries? *Addict Abingdon Engl* 2014;**109**:1081–90. doi:10.1111/add.12571

55 Ministère de l'Industrie, du Commerce et de l'Artisanat. Communiqué MICA. https://www.commerce.gov.bf/accueil (accessed 3 Mar 2021).

56 Burkina24. Production et commercialisation d'alcool au Burkina : Bientôt un cahier de charges. https://www.burkina24.com/2020/07/02/production-et-commercialisation-dalcool-au-burkina-bientot-un-cahier-de-charges/

57 Roerecke M. On bias in alcohol epidemiology and the search for the perfect study. *Addict Abingdon Engl* 2017;**112**:217–8. doi:10.1111/add.13549

For peer review only - http://bmjopen.bmj.com/site/about/guidelines.xhtml

Tables

Table 1: Definition of recoded exposure variables.

Variables	Categories
Age groups	"25 to 34 years old", "35 to 44 years old", "45 to 54 years old", "55 to 64 years old"
Education	"None", "Primary", "Secondary", "Tertiary"
Marital status	"Single", "Married", "Divorced/widowed"
Occupational status	"Wage earner", "Self-employed", "Jobless", "Housemaker"
Alcohol consumption (WHO recommendations) [37]	None: Never intake of alcohol<br>Low: intake of an average quantity of pure alcohol of less than 40 g per day for men and less than 20 g for women<br>Mid: corresponds to taking an average quantity of pure alcohol of between 40 g and 59.9 g per day for men and between 20 g and 39.9 g for women<br>Abusive: intake of an average quantity of pure alcohol greater than or equal to 60 g per day for men and greater than or equal to 40 g for women.

Note than one glass of alcohol (beer, wine, 'dolo') contains 10g of ethanol, the recall period is 30 days.

For peer review only - http://bmjopen.bmj.com/site/about/guidelines.xhtml

Table 2: Socio-demographic characteristics of the study sample and prevalence of alcohol and abusive alcohol consumption

Variables	Participants, (n)	Low consumption [95% CI]	Mid consumption [95% CI]	Abusive consumption, % [95% CI]
**Age groups, years (n= 4,692)**				
25 to 34 years	2,124	10.6 [8.4 – 13.1]	7.0 [5.5 – 8.8]	1.9 [1.2 – 2.9]
35 to 44 years	1,181	13.1 [10.4 – 16.3]	8.9 [6.8 – 11.5]	3.1 [1.9 – 4.9]
45 to 54 years	841	14.8 [11.0 – 19.6]	9.5 [7.1 – 12.5]	3.8 [2.3 – 6.1]
55 to 64 years	546	18.3 [14.1 – 23.4]	11.3 [8.3 – 15.3]	3.0 [1.5 – 6.2]
**Sex (n = 4,692)**				
Female	2,436	8.6 [6.3 – 11.7]	10.8 [8.8 – 13.3]	0.9 [0.6 – 1.5]
Male	2,256	18.1 [15.5 – 21.1]	5.8 [4.6 – 7.1]	4.8 [3.4 – 6.8]
**Living environment (n = 4,692)**				
Urban	945	12.7 [8.4 – 18.6]	10.0 [7.3 – 13.5]	4.3 [2.5 – 7.2]
Rural	3,747	13.1 [10.8 – 15.6]	8.0 [6.4 – 9.9]	2.2 [1.5 – 3.1]
**Education (n = 4,684)**				
None	3,622	12.2 [9.9 – 14.8]	7.8 [6.2 – 9.7]	2.0 [1.4 – 2.9]
Primary	728	15.6 [11.9 – 20.1]	8.8 [6.7 – 11.5]	3.6 [2.0 – 6.3]
Secondary	334	15.7 [11.7 – 20.7]	15.3 [11.2 – 20.4]	8.2 [4.5 – 14.6]
**Marital status (n = 4,688)**				
Single	333	16.9 [11.8 – 23.6]	9.2 [6.1 – 13.8]	5.2 [2.6 – 10.1]
Married	4,043	12.8 [10.6 – 15.4]	8.2 [6.7 – 9.9]	2.4 [1.8 – 3.3]
Divorced/Widowed	311	10.4 [6.9 – 9.9]	12.5 [8.8 – 17.6]	3.8 [1.6 – 8.5]
**Occupational status (n = 4,692)**				

For peer review only

Wage earner	281	15.1 [10.7 – 20.9]	12.7 [8.5 – 18.5]	9.9 [5.8 – 16.2]
Self-employed	3,249	13.2 [11.2 – 15.5]	7.9 [6.3 – 9.8]	2.9 [2.1 – 4.3]
Jobless	125	11.8 [6.7 – 20.0]	10.4 [5.7 – 18.2]	0.5 [0.1 – 3.4]
Housemaker	1,037	11.9 [7.6 – 18.2]	8.9 [6.8 – 11.6]	0.4 [0.1 – 1.4]
**Tobacco consumption (n = 4,692)**				
No	4,217	12.1 [10.0 – 14.6]	8.4 [6.9 – 10.1]	2.1 [1.6 – 2.9]
< 5 cigs/day	210	23.9 [17.6 – 31.5]	1.9 [0.8 – 4.5]	6.4 [3.2 – 12.6]
5 – 10 cigs/day	184	19.8 [13.5 – 28.1]	13.8 [8.5 – 21.4]	6.6 [3.2 – 13.0]
> 10 cigs/day	81	18.4 [10.7 – 29.8]	17.9 [9.8 – 30.1]	15.5 [4.6 – 40.7]

For peer review only

Table 3: Factors associated with alcohol and abusive alcohol consumptions in population aged 25 – 64 years.

Variables	Low consumption		Mid consumption		Abusive consumption	
	n	AOR [95% CI]	n	AOR [95% CI]	n	AOR [95% CI]
**Age groups**						
25 years to 34 years (n = 2,224)	221	Ref	151	Ref	41	Ref
35 years to 44 years (n = 1,181)	161	**1.46 [1.09 – 1.95]***	112	**1.55 [1.14 – 2.12]****	30	**1.86 [1.06 – 3.27]***
44 years to 54 years (n = 841)	123	**1.79 [1.34 – 2.40]*****	81	**1.81 [1.25 – 2.62]****	30	**2.67 [1.36 – 5.25]****
55 to 64 (n =546)	105	**2.36 [1.59 – 3.51]*****	55	**2.50 [1.54 – 4.07]*****	19	**2.37 [1.01 – 5.92]***
**Sex**						
Female (n = 2,436)	204	Ref	270	Ref	22	Ref
Male (n = 2,256)	406	**2.68 [1.96 – 3.67]*****	129	**0.38 [0.26 – 0.55]*****	98	**2.89 [1.56 – 2.37]****
**Living environment**						
Urban (n = 945)	111	Ref	109	Ref	30	Ref
Rural (n = 3,747)	499	1.18 [0.59 – 2.33]	290	1.12 [0.68 – 1.87]	90	0.76 [0.40 – 1.45]

For peer review only - http://bmjopen.bmj.com/site/about/guidelines.xhtml

**Education**						
None (n = 3,622)	452	Ref	281	Ref	81	Ref
Primary (n = 728)	112	**1.47 [1.05 – 2.06]***	67	1.47 [0.99 – 2.18]	19	1.60 [0.81 – 3.18]
Secondary (n = 334)	46	**2.08 [1.07 – 4.03]***	49	**2.97 [1.48 – 5.92]****	19	**3.50 [1.34 – 9.15]****
**Marital status**						
Single (n = 333)	57	Ref	31	Ref	17	Ref
Married (n = 4,043)	514	0.66 [0.40 – 1.10]	93	0.74 [0.43 – 1.30]	93	0.56 [0.23 – 1.36]
Divorced/Widowed (n = 311)	39	0.56 [0.29 – 1.08]	10	0.83 [0.42 – 1.63]	10	1.01 [0.31 – 3.33]
**Occupational status**						
Wage earner (n = 281)	47	Ref	35	Ref	20	Ref
Self-employed (n = 3,249)	436	1.10 [0.58 – 2.06]	246	0.90 [0.53 – 1.51]	95	0.65 [0.26 – 1.67]
Jobless (n = 125)	16	0.75 [0.37 – 1.53]	15	0.77 [0.33 – 1.76]	1	**0.05 [0.01 – 0.44]****
House maker (n = 1,037)	111	2.20 [0.95 – 5.08]	103	0.74 [0.41 – 1.33]	4	0.23 [0.04 – 1.15]
**Tobacco consumption**						
No (n = 4,217)	512	Ref	354	Ref	86	Ref

For peer review only - http://bmjopen.bmj.com/site/about/guidelines.xhtml

< 5 cigs/day (n = 210)	47	**1.72 [1.13 – 2.62]***	6	0.52 [0.21 – 1.31]	14	**2.42 [1.07 – 5.63]***
5 – 10 cigs/day (n = 184)	36	1.60 [0.98 – 2.62]	23	**4.20 [2.25 – 7.83]*****	11	**2.48 [1.06 – 5.79]***
> 10 cigs/day (n = 81)	15	1.77 [0.83 – 3.75]	16	**6.08 [2.75 – 13.4]*****	9	**6.58 [1.96 – 22.11]****

* p = 0.05, ** p = 0.01, *** p < 0.001

For peer review only

Table 4: Factors associated with abusive alcohol consumptions in population aged 25 – 64 years consuming alcohol.

Variables	Alcohol consumption	Abusive alcohol consumption	
	N	N	AOR [95%CI]
**Age groups**			
25 years to 34 years	413	41	Ref
35 years to 44 years	303	30	1.55 (0.84 – 2.86)
44 years to 54 years	234	30	1.91 (0.95 – 3.85)
55 to 64	179	19	0.99 (0.38 – 2.57)
**Sex**			
Female	496	22	Ref
Male	633	98	**2.53 (1.38 – 4.68)****
**Living environment**			
Urban	250	30	Ref
Rural	879	90	0.68 (0.34 – 1.36)
**Education**			
None	814	81	Ref
Primary	198	19	0.89 (0.43 – 1.86)
Secondary	114	19	1.25 (0.48 – 3.25)
**Marital status**			
Single	105	17	Ref
Married	930	93	0.70 (0.32 – 1.53)
Divorced/Widowed	94	10	1.41 (0.45 – 4.46)
**Occupational status**			
Wage earner	102	20	Ref
Self-employed	777	95	0.51 (0.19 – 1.50)
Jobless	32	1	**0.05 (0.01 – 0.47)****
House maker	218	4	**0.14 (0.02 – 0.78)***
**Tobacco consumption**			
No	952	86	Ref
< 5 cigs/day	67	14	1.71 (0.68 – 4.26)

For peer review only - http://bmjopen.bmj.com/site/about/guidelines.xhtml

5 – 10 cigs/day	70	11	1.20 (0.51 – 2.86)
> 10 cigs/day	40	9	2.26 (0.63 – 8.05)

* p = 0.05, ** p = 0.01, *** p < 0.001

For peer review only

For peer review only - http://bmjopen.bmj.com/site/about/guidelines.xhtml

Table 5: Risk of alcohol consumption in the different regions of the country.

Region	N	Alcohol consumption	
		n	AOR (95%CI)
Centre	548	168	Ref
Boucle du Mouhoun	469	147	1.23 [0.63 – 2.39]
Cascades	166	45	0.58 [0.28 – 1.19]
Centre-Est	398	72	0.51 [0.24 – 1.06]
Centre-Nord	434	87	0.67 [0.35 – 1.29]
Centre-Ouest	390	121	0.94 [0.45 – 1.99]
Centre-Sud	217	51	0.66 [0.28 – 1.54]
Est	376	63	0.40 [0.22 – 0.74]**
Haut-Bassin	509	89	0.39 [0.24 – 0.66]***
Nord	420	72	0.66 [0.28 – 1.58]
Plateau Central	236	62	1.01 [0.37 – 2.75]
Sahel	315	4	0.02 [0.01 – 0.15]***
Sud-Ouest	214	148	5.63 [1.98 – 16.01]***

Model is adjusted for age, sex, living environment, education, marital status, occupational status and tobacco consumption. Alcohol consumption includes the different levels of consumption

* p = 0.05, ** p = 0.01, *** p < 0.001

For peer review only

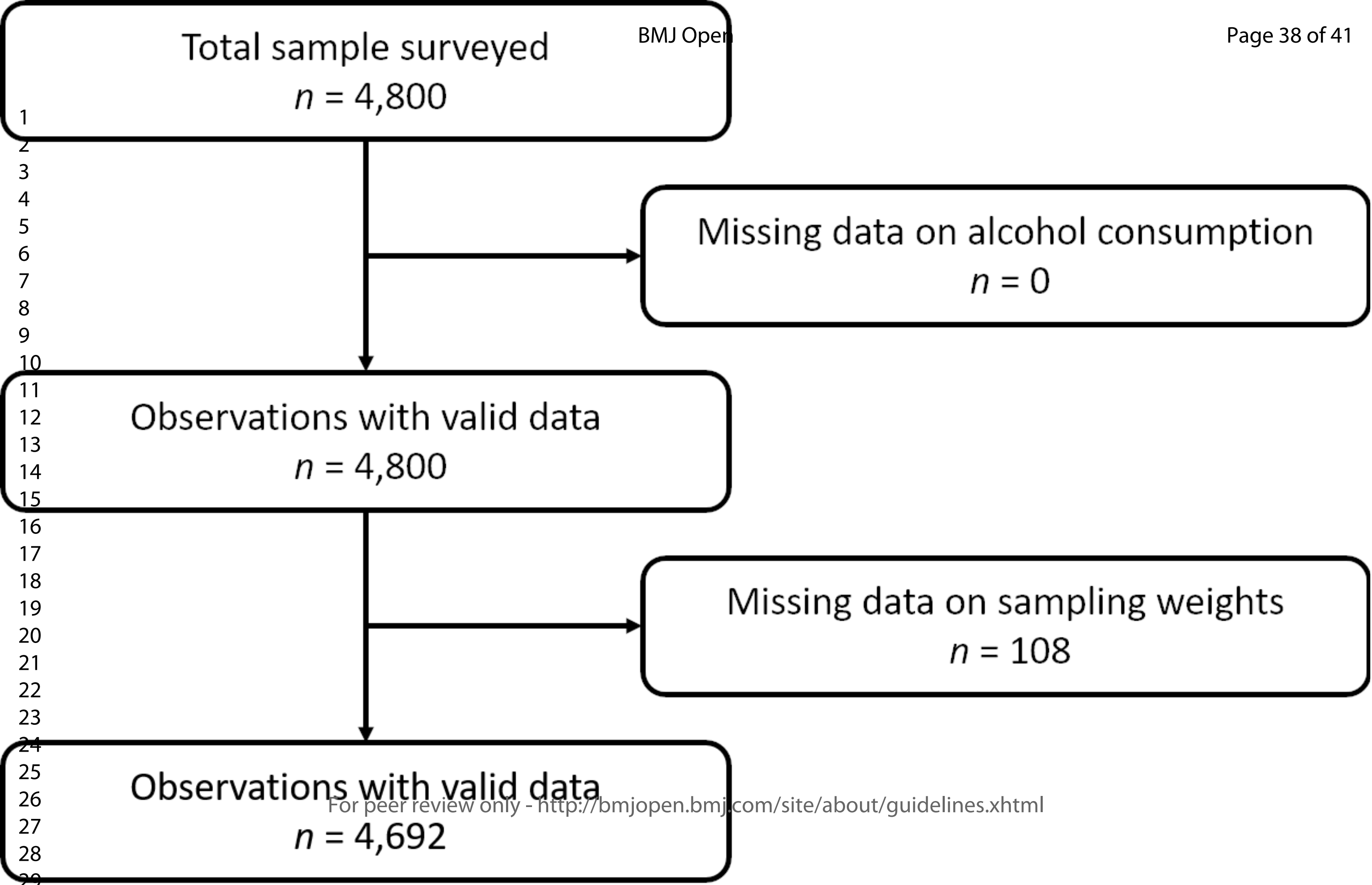

For peer review only - http://bmjopen.bmj.com/site/about/guidelines.xhtml

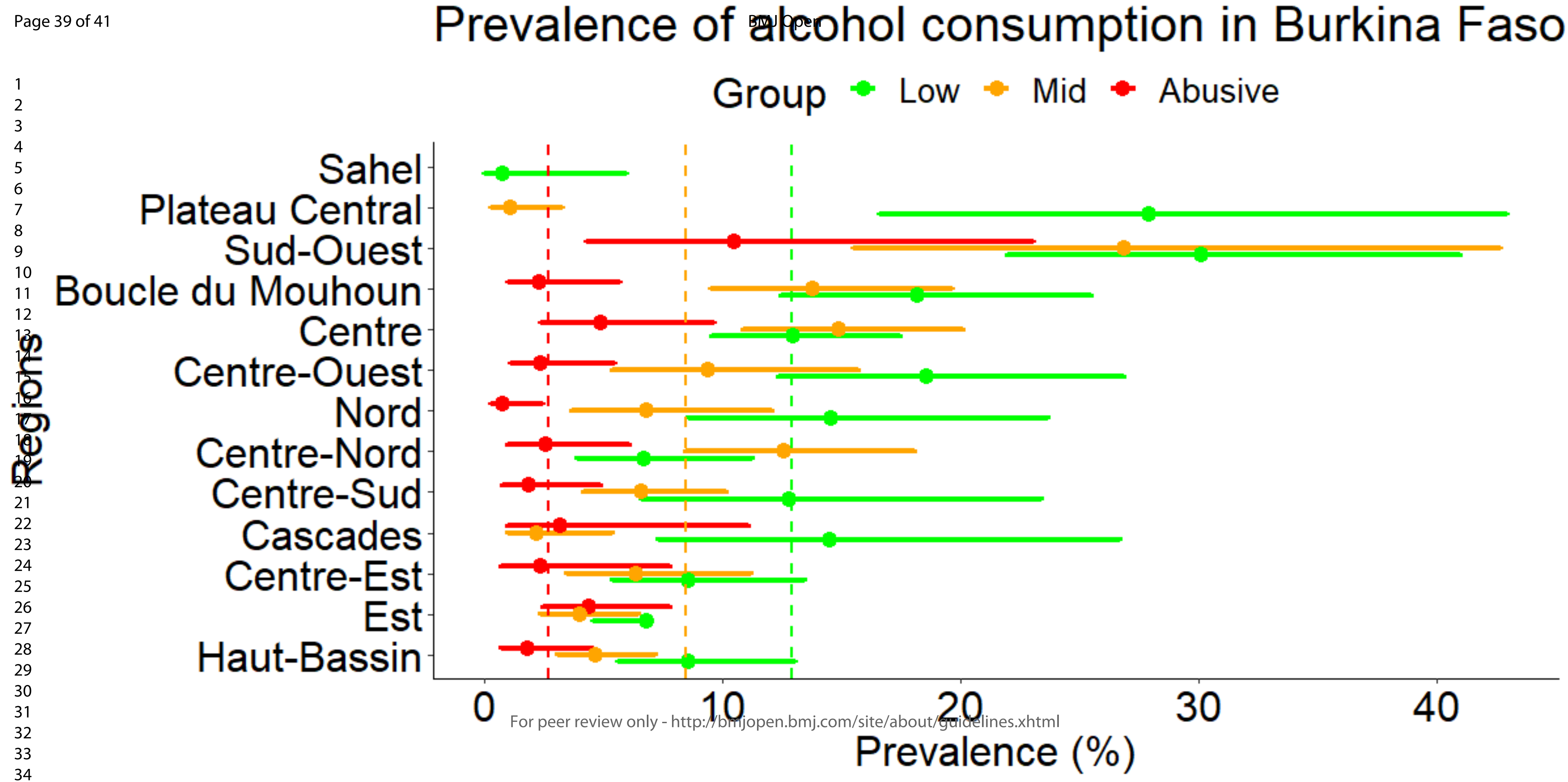
Prevalence of alcohol consumption in Burkina Faso
Group Low Mid Abusive
Regions
Sahel
Plateau Central
Sud-Ouest
Boucle du Mouhoun
Centre
Centre-Ouest
Nord
Centre-Nord
Centre-Sud
Cascades
Centre-Est
Est
Haut-Bassin
0
10
20
30
40
Prevalence (%)

STROBE Statement—Checklist of items that should be included in reports of ***cross-sectional studies***

	Item No	Recommendation	Page
**Title and abstract**	1	(*a*) Indicate the study's design with a commonly used term in the title or the abstract	Yes
		(*b*) Provide in the abstract an informative and balanced summary of what was done and what was found	Yes
**Introduction**			
Background/rationale	2	Explain the scientific background and rationale for the investigation being reported	p.3
Objectives	3	State specific objectives, including any prespecified hypotheses	p.4
**Methods**			
Study design	4	Present key elements of study design early in the paper	Data source on p.5-6, sampling p.6
Setting	5	Describe the setting, locations, and relevant dates, including periods of recruitment, exposure, follow-up, and data collection	p.6
Participants	6	(*a*) Give the eligibility criteria, and the sources and methods of selection of participants	STEPs survey defined by WHO (p.6)
Variables	7	Clearly define all outcomes, exposures, predictors, potential confounders, and effect modifiers. Give diagnostic criteria, if applicable	p.7 and Table 1 and 2
Data sources/ measurement	8*	For each variable of interest, give sources of data and details of methods of assessment (measurement). Describe comparability of assessment methods if there is more than one group	NA
Bias	9	Describe any efforts to address potential sources of bias	NA
Study size	10	Explain how the study size was arrived at	p.5-6
Quantitative variables	11	Explain how quantitative variables were handled in the analyses. If applicable, describe which groupings were chosen and why	Definition of recoded exposure variables is presented in Table 1
Statistical methods	12	(*a*) Describe all statistical methods, including those used to control for confounding	p.7-8
		(*b*) Describe any methods used to examine subgroups and interactions	Multivariable logistic regression models and adjusted OR have been used (p.8 and in the results section)
		(*c*) Explain how missing data were addressed	Flow of the study participants is presented in Figure 1
		(*d*) If applicable, describe analytical methods taking account of sampling strategy	All data were weighted according to the cluster sampling design of the survey using strata and primary

For peer review only - http://bmjopen.bmj.com/site/about/guidelines.xhtml

			sampling units to allow the sample to be nationally representative (p.6)
		(*e*) Describe any sensitivity analyses	NA
**Results**			
Participants	13*	(a) Report numbers of individuals at each stage of study—eg numbers potentially eligible, examined for eligibility, confirmed eligible, included in the study, completing follow-up, and analysed	Figure 1
		(b) Give reasons for non-participation at each stage	NA
		(c) Consider use of a flow diagram	Figure 1
Descriptive data	14*	(a) Give characteristics of study participants (eg demographic, clinical, social) and information on exposures and potential confounders	Table 2
		(b) Indicate number of participants with missing data for each variable of interest	Figure 1
Outcome data	15*	Report numbers of outcome events or summary measures	Table 3
Main results	16	(*a*) Give unadjusted estimates and, if applicable, confounder-adjusted estimates and their precision (eg, 95% confidence interval). Make clear which confounders were adjusted for and why they were included	Table 3
		(*b*) Report category boundaries when continuous variables were categorized	Table 1
		(*c*) If relevant, consider translating estimates of relative risk into absolute risk for a meaningful time period	NA
Other analyses	17	Report other analyses done—eg analyses of subgroups and interactions, and sensitivity analyses	Multivaiate analysis are presented in Table 3 and explained in the method p.6-8
**Discussion**			
Key results	18	Summarise key results with reference to study objectives	First paragraphs of the discussion (p.11)
Limitations	19	Discuss limitations of the study, taking into account sources of potential bias or imprecision. Discuss both direction and magnitude of any potential bias	Start of the discussion (p.11)
Interpretation	20	Give a cautious overall interpretation of results considering objectives, limitations, multiplicity of analyses, results from similar studies, and other relevant evidence	p.11-12
Generalisability	21	Discuss the generalisability (external validity) of the study results	p.12-13
**Other information**			

For peer review only

Funding	22	Give the source of funding and the role of the funders for the present study and, if applicable, for the original study on which the present article is based	None

*Give information separately for exposed and unexposed groups.

Note: An Explanation and Elaboration article discusses each checklist item and gives methodological background and published examples of transparent reporting. The STROBE checklist is best used in conjunction with this article (freely available on the Web sites of PLoS Medicine at http://www.plosmedicine.org/, Annals of Internal Medicine at http://www.annals.org/, and Epidemiology at http://www.epidem.com/). Information on the STROBE Initiative is available at www.strobe-statement.org.

For peer review only

BMJ Open

Alcohol consumption and associated risk factors in Burkina Faso: results of a population-based cross-sectional survey.

Journal:	*BMJ Open*
Manuscript ID	bmjopen-2021-058005.R2
Article Type:	Original research
Date Submitted by the Author:	18-Jan-2022
Complete List of Authors:	Bonnechère, Bruno; Hasselt University, REVAL Rehabilitation Research Center<br>Samadoulougou, Sékou; Quebec Heart and Lung Institute Research Center; Laval University<br>Cisse, Kadari; Institut de Recherche en Sciences de la Sante, Biomedical et santé publique; Université Libre de Bruxelles- Ecole de santé publique, Centre de Recherche en Epidémiologie, Biostatistiques et Recherche Clinique<br>Tassembedo, Souleymane; Centre MURAZ, Programme de Recherche sur les maladies Infectieuses<br>Kouanda, Seni; Institut de Recherche en Sciences de la Sante<br>Kirakoya-Samadoulougou, Fati; Université Libre de Bruxelles, Biostatistics, and Clinical Research
**Primary Subject Heading**:	Epidemiology
Secondary Subject Heading:	Addiction, Global health, Health policy, Public health
Keywords:	EPIDEMIOLOGY, PUBLIC HEALTH, TOXICOLOGY

SCHOLARONE™
Manuscripts

I, the Submitting Author has the right to grant and does grant on behalf of all authors of the Work (as defined in the below author licence), an exclusive licence and/or a non-exclusive licence for contributions from authors who are: i) UK Crown employees; ii) where BMJ has agreed a CC-BY licence shall apply, and/or iii) in accordance with the terms applicable for US Federal Government officers or employees acting as part of their official duties; on a worldwide, perpetual, irrevocable, royalty-free basis to BMJ Publishing Group Ltd ("BMJ") its licensees and where the relevant Journal is co-owned by BMJ to the co-owners of the Journal, to publish the Work in this journal and any other BMJ products and to exploit all rights, as set out in our licence.

The Submitting Author accepts and understands that any supply made under these terms is made by BMJ to the Submitting Author unless you are acting as an employee on behalf of your employer or a postgraduate student of an affiliated institution which is paying any applicable article publishing charge ("APC") for Open Access articles. Where the Submitting Author wishes to make the Work available on an Open Access basis (and intends to pay the relevant APC), the terms of reuse of such Open Access shall be governed by a Creative Commons licence – details of these licences and which Creative Commons licence will apply to this Work are set out in our licence referred to above.

Other than as permitted in any relevant BMJ Author's Self Archiving Policies, I confirm this Work has not been accepted for publication elsewhere, is not being considered for publication elsewhere and does not duplicate material already published. I confirm all authors consent to publication of this Work and authorise the granting of this licence.

For peer review only

Alcohol consumption and associated risk factors in Burkina Faso: results of a population-based cross-sectional survey

For peer review only

Bonnechère Bruno[1*], Samadoulougou Sékou[2,3], Cissé Kadari[4,5], Tassembedo Souleymane[6], Kouanda Seni[5,7], Kirakoya-Samadoulougou Fati[4]

[1] REVAL Rehabilitation Research Center, Faculty of Rehabilitation Sciences, Hasselt University, Diepenbeek, Belgium

[2] Evaluation Platform on Obesity Prevention, Quebec Heart and Lung Institute Research Center, Quebec City, QC G1V 4G5, Canada

[3] Centre for Research on Planning and Development (CRAD), Laval University, Quebec, G1V 0A6, Canada

[4] Centre de Recherche en Epidémiologie, Biostatistiques et Recherche Clinique, Ecole de Santé Publique, Université libre de Bruxelles, Brussels, Belgium

[5] Institut de recherche en sciences de la santé (IRSS), Ouagadougou, 03 BP 7102, Burkina Faso

[6] Département de recherche clinique, centre Muraz, Bobo-Dioulasso, Burkina Faso

[7] Institut Africain de Santé publique (IASP), Ouagadougou, 12 BP 199, Burkina Faso

* corresponding author : bruno.bonnechere@uhasselt.be

Prof. Dr. Bonnechère Bruno

Agoralaan Gebouw A

3590 Diepenbeek

Belgium

For peer review only - http://bmjopen.bmj.com/site/about/guidelines.xhtml

Word count: 3681

Tables: 5

Figures: 2

For peer review only

For peer review only - http://bmjopen.bmj.com/site/about/guidelines.xhtml

Abstract

Objectives: Lifestyle modifiable risk factors are a leading preventable cause of non-communicable diseases (NCDs), with alcohol consumption among the most important. Studies characterizing the prevalence of alcohol consumption in low-income countries are lacking. This study describes the prevalence of different levels of alcohol consumptions in Burkina Faso and its associated factors.

Design: Data from the 2013 Burkina Faso World Health Organization (WHO) Stepwise approach to Surveillance (STEPS) survey were analyzed. The prevalence of alcohol consumption over the last 30 days was recorded into categories according to WHO recommendations: low, mid, or abusive alcohol consumption. Multinomial logistic regression analyses identified factors associated with the different levels of alcohol consumption use.

Setting: Population-based cross-sectional survey in Burkina Faso.

Participants: 4692 participants of both sexes aged 25 to 64 years were included in the study.

Results: In the whole sample 3,559 participants (75.8% [72.5 – 78.7]) were not consuming any alcohol, 614 (12.9% [10.9 – 15.3]) had a low alcohol consumption, 399 (8.5% [7.1 – 10.1]) a mid alcohol consumption and 120 (2.7% [2.0 – 3.7]) an abusive consumption. Age was associated with alcohol intake with a gradient effect, older people having a higher level of consumptions (AOR: 2.36, 95%CI [1.59 – 3.51] for low consumption, 2.50 [1.54 – 4.07] for mid consumption and 2.37 [1.01 – 5.92] for abusive consumption in comparison with no consumption). Tobacco consumption was also significantly associated with alcohol intake with a gradient effect, those with higher tobacco consumption being at higher risk of abusive

For peer review only - http://bmjopen.bmj.com/site/about/guidelines.xhtml

alcohol intake (AOR = 6.08 [2.75 – 13.4] for moderate consumption and 6.58 [1.96 – 22.11] abusive consumption).

Conclusion: Our data showed an important burden of alcohol consumption in Burkina Faso, which varied with age and tobacco use. To effectively reduce alcohol consumption in Burkina Faso, comprehensive control and prevention campaigns should consider these associated factors.

Keywords

Alcohol consumption; Abusive alcohol consumption; Burkina Faso; Risk factors; Surveillance; WHO STEPS survey

For peer review only - http://bmjopen.bmj.com/site/about/guidelines.xhtml

Article summary

Strengths and Limitations of this study

- To the best of our knowledge, this study is the first national representative study on alcohol consumption within the adult population of Burkina Faso.
- This study presents alcohol consumption level and associated risk factors (age and tobacco consumption) in Burkina Faso using a large representative cohort of 4672 participants.
- The main limitation is that alcohol consumptions and its associated risk factors were obtained during interviews (memory and social desirability biases).
- Another limitation of this study is that we used data from 2013 (latest results available at large scale) and that the prevalence may have change due to the recent implementation of alcohol policies to limits the consumption.

For peer review only

For peer review only - http://bmjopen.bmj.com/site/about/guidelines.xhtml

Background

The increase in life expectancy [1] combined with unhealthy behaviors and physical inactivity [2] are linked to a rise in non-communicable diseases (NCDs) such as cardiovascular disease, cancer, chronic respiratory disease, and diabetes [3]. Currently, the leading causes of ill health in the world, NCDs account for seven out of ten deaths worldwide [4].

Low and mid-income countries are facing the emergence of NCDs [5]. Although some risk factors have been identified such as high blood pressure [6], lack of physical activity [7], inadequate diet [8], etc., there is still a lack of comprehensive data on other modifiable risk factors, in particular tobacco and alcohol consumption [9], in Burkina Faso.

Recently, clear evidence of increased alcohol consumption and attributable harm in many low and mid-income countries has been highlighted [10]. Furthermore, it is predicted to have a more harmful effect if an effective policy is not adopted [11]. Currently, the prevalence of alcohol intake among the African adult population is about 30% [12], which is lower compared to the rest of the world where 40% of the world's adult population consumes alcohol and the average consumption per drinker is 17.1 liters per year. Interestingly, the prevalence of lifetime abstention, the level of alcohol consumption, and the drinking patterns vary widely across regions of the world [13]. For example, Eastern Europe and Southern Africa had the most detrimental pattern of drinking scores. At the same time, Europe (Eastern and Central) and sub-Saharan Africa (Southern and West) are the most important consumers of alcohol [13]. In Africa, among current drinkers, the prevalence of heavy drinking varied between 7% and 77%, and the prevalence of daily light drinkers varied between 0% and 21%. Overall,

For peer review only - http://bmjopen.bmj.com/site/about/guidelines.xhtml

drinking patterns varied significantly between and within the examined African countries [14], more recent studies showed the same trends in South Africa and Ethiopia [15,16].

One of the major current concerns related to alcohol consumption in Africa is that alcohol companies have targeted Africa as a new market. With the expected increases in the number of potential new alcohol consumers, especially young people, and women, the African continent has indeed been identified by the alcohol beverage industry and market researchers as a key area for alcohol market growth [17,18]. It is, therefore, of particular importance to identify subjects the most at risk of alcohol consumption. Currently, two major risk factors have been identified worldwide: gender with the male being more at risk [19] and the socioeconomic level where the effect is less clear as the associations between socioeconomic disadvantage and heavier drinking vary depending on country-level income [20]. Pregnant women are particularly more vulnerable due to the fetus's susceptibility [21–24].

Another important aspect highlighting the need for local studies is that it has been shown that there are significant disparities in alcohol use between Africans (Ghanaians) residing in Europe and Africa, indicating that migration has a significant impact on drinking habits and also implying that alcohol reduction initiatives may need distinct techniques.[25]

There is currently a lack of information about the prevalence of alcohol consumption in Burkina Faso and the associated risk factors. Differences in cultural factors (e.g., beliefs and practices) may influence health status, but social, economic, and structural determinants of health during people's lifespans appear to be associated with health inequities between ethnic groups as well. Therefore, cultural influences should not be overemphasized as discrete explanatory factors for health inequities [26].

For peer review only - http://bmjopen.bmj.com/site/about/guidelines.xhtml

A first national survey was conducted in 2013 using the WHO STEPwise approach to risk factors surveillance (STEPS). The STEPS survey is a simple, standardized method for collecting, analyzing, and disseminating data in WHO member countries. It covered a representative sample of the adult population. The first analyses performed were about the evaluation of cardiovascular risk [27]. In a previous study, we show that tobacco consumption was highly correlated with alcohol consumption in men [28]. Therefore, in this study we aimed to investigate the prevalence of alcohol consumption and its associated risk factors. Such analyses are needed to drive more efficient prevention campaigns for both alcohol and tobacco consumption.

Methods

Study settings

Burkina Faso is a landlocked country in West Africa of 272 967.47 km^2. The country is divided into 13 administrative regions and is limited in the North and West by Mali, in the East by Niger, and in the South by Benin, Togo, Ghana, and Côte d'Ivoire.

In 2020, the population was estimated at 21,510,181 inhabitants [29]. The majority of the population (77.30%) lives in rural areas and relies on agriculture and livestock as the main source of income [30]. The median age of the population is 17.9 years old. In 2018, the total fertility rate was 5.2 and the life expectancy at birth was 62.3 years in 2019 [31]. Like most low-income countries, Burkina Faso must face the double burden of infectious and chronic diseases. The country is regularly confronted with outbreaks such as measles, cerebrospinal

For peer review only - http://bmjopen.bmj.com/site/about/guidelines.xhtml

meningitis and, malaria [32], while NCDs constitute a rising public health problem with only limited financial resources allocated.

In Burkina Faso, several sources of alcohol manufacturing exist modern brewery and legally imported alcohol, prohibited alcohol mostly imported from neighboring countries and traditional beer manufacturers. Burkina Faso is a secular country with more than half of the population declared as being of Islamic faith (61.6%), 22.5% Christians, and 15.4% traditional religions [33].

Harmful alcohol use and its adverse events (i.e., road traffic accidents, domestic violence) are however important and raising public health concerns in Burkina Faso [34].

Study design

This is an analytical cross-sectional study of the WHO STEPS survey conducted in Burkina Faso in 2013. Complete details on the methodology and sampling procedure were described in the WHO STEPS 2013 Burkina report [35], and in previous studies using this database [27,28,36]. The participants were selected using a three-stage cluster sampling process described in the sampling method.

Study population

Participants of the study were adults of both sexes aged 25 to 64 years old who had been living in Burkina Faso for at least six months. Exclusion criteria were: people with disabilities hampering their ability to answer the questions (e.g., intellectual disabilities, serious mental disorders, cognitive impairment, etc.).

For peer review only - http://bmjopen.bmj.com/site/about/guidelines.xhtml

Patient and public involvement

No patients were involved in the study design or analysis of the anonymous data set.

Sampling

The STEPS survey of Burkina Faso was conducted on a representative sample of 4,800 individuals, the response rate of the STEPS survey was 99.1% in Burkina Faso [32]. After excluding participants with missing information about sampling weight, 4,692 individuals were included in the present analysis. The diagram flow of study participants is presented in Figure 1. A three levels sampling frame was performed. In the first stage, geographic areas were stratified into rural and urban, and EAs were selected with probability proportional to their size from both strata. A total of 240 EAs were selected: 185 from rural areas and 55 from urban areas. In the second stage, 20 households were selected from each EA. In the third stage, one person aged from 25 to 64 years in each household was selected using the Kish method.

Data collection

A full description of the study design and the data collection has been published elsewhere [27,36]. Briefly, all data on alcohol consumption were collected using a standardized questionnaire during face-to-face interviews in the language most spoken by the respondent (Step 1 of the STEP survey). The questionnaires were pre-tested on the field before being used for the national survey. Alcohol consumption was assessed with the question '*Have you ever consumed any alcohol such as beer, wine, spirits or dolo*'? If the answer is yes, the current alcohol consumption was assessed with the questions '*During the past 30 days, how many days did you consume alcohol drinks?' and 'When you are drinking, what is the average number of drinks* ?'. Four levels were defined: no consumption, low, mid, and abusive

For peer review only - http://bmjopen.bmj.com/site/about/guidelines.xhtml

For peer review only

consumption (see Table 1 for WHO definition). According to the WHO recommendation, the level defining the different levels of consumption have been defined differently for males and females.

Other information such as demographic information, anthropometric and behavioral measurements were also collected (Step 2). In our study, all the variables used in the manuscript were collected in Step I.

Study variables

The dependent variable was the mean alcohol consumption in the last 30 days. First, we analyzed alcohol consumption to define the profile of the drinker.

The independent variables were: age, sex, education, marital status, occupational status, and tobacco consumption (see Table 1 for the definition of the variables).

Data analysis

Some independent quantitative variables were transformed into categorical variables. The list of the variables with the recoded variables is presented in Table 1. Categorical variables were described using proportions. The Chi-squared test was used in univariable analysis to test the association between the outcome (the levels of alcool consumption) and the categorical variables (age, sex, living environment, education, marital status, occupational status, tobacco consumption). We first analyzed the profile and levels of alcohol consumption using multinomial logistic regression defining no alcohol consumption as the reference value. Then we identify the profile of the abusive consumer, following WHO recommendations [37], amongst current drinkers using logistic regression grouping low and mid consumption as reference group.

For peer review only - http://bmjopen.bmj.com/site/about/guidelines.xhtml

Adjusted Odds Ratios (AORs) were calculated for the studied variables and presented with 95% confidence intervals. We then analyzed discrepancies between the different regions using adjusted AORs for the different regions.

All the analyses were carried out considering the sampling weight and the sampling design. Statistical analyses were performed at an overall significance level of 0.05. Statistics have been conducted in STATA (version 13) and RStudio (version 1.1.442) with R version 3.4.4).

Results

Characteristics of the population are presented in Table 2. Most of the participants were rural residents (79.7%). The population was predominantly young, with the 25-34 years group representing 41.9%. Women were more represented, with 54.3% of the total sample.

In the whole population 3,559 (75.8% [72.5 – 78.7]) were not consuming any alcohol, 614 (12.9% [10.9 – 15.3]) had a low alcohol consumption, 399 (8.5% [7.1 – 10.1]) a mid alcohol consumption and 120 (2.7% [2.0 – 3.7]) an abusive consumption. There are important differences between the different regions of the countries; the results are presented in Figure 2. In the *Sahel* region, the prevalence is null while in the Sud-Ouest regions the prevalence for all the levels is statistically significantly higher than the country's average values.

The prevalence of abusive consumption was not different in urban setting and rural setting (4.3 % [2.5 – 7.2] versus 2.1% [1.5 – 3.1], p = .19). The repartition of consumption was statistically significantly different between urban and rural environment (*p* =.001): There was a higher proportion of low alcohol consumption is the rural area (13.1% compared to 12.7%) while in the urban area there is more mid (10.0% Vs. 8.0%) and high consumption (4.3% Vs. 2.2%).

For peer review only - http://bmjopen.bmj.com/site/about/guidelines.xhtml

For peer review only

In multivariable analysis (Table 3), first concerning the alcohol consumption, there is a statistically significant gradient effect of the age regardless of the level of consumption. We found that compared to no alcohol consumption, the odds of engaging in low alcohol consumption was 1.46 times higher among people with 35-44 years compared to those with 25-34 years old (AOR = 1.46 [1.09 – 1.95]). The odds were 1.76 times higher (AOR: 1.76 [1.34 – 2.40]) and 2.36 [1.59 – 3.51] for the 45-54 and 55-64 years old respectively compared to the 25-34 years old group. Regarding mid consumption, the odds were 1.55 [1.14 – 2.12], 1.81 [1.25 – 2.62], and 2.50 [1.54 – 4.07] times higher for 35-44 years, 45-54 years and 55-64 years respectively compared to 25-34 years old. Similar results were found when considering abusive alcohol consumption (AOR = 1.86 [1.06 – 3.27], 2.67 [1.36 – 5.25], 2.37 [1.01 – 5.92] for abusive consumption. Interestingly, the effect of gender is different regarding the level of consumption: there is an increased risk in male for low (AOR = 2.68 [1.96 – 3.67]) and abusive consumption (AOR = 2.89 [1.56 – 2.37]), while there is a protective effect against moderate consumption (AOR = 0.38 [0.26 – 0.55]) compared to female. The level of education is also associated with an increased risk, regardless of consumption level (AOR = 2.08 [1.07 – 4.03], 2.97 [1.48 – 5.92], and 3.50 [1.34 – 9.15] for low, mid, and abusive consumption respectively for participants who reached secondary levels in comparison of people without any education).

There is also an important gradient effect associated with tobacco consumption: the consumption of small tobacco quantity (<5 cigs/day) is associated with an increased risk of low alcohol consumption (AOR = 1.72 [1.13 – 2.62]) while the consumption of high quantity of tobacco is associated with mid and abusive alcohol consumption (AOR = 6.08 [2.75 – 13.4] and 6.58 [1.96 – 22.11] respectively).

For peer review only - http://bmjopen.bmj.com/site/about/guidelines.xhtml

Finally, we computed the risk of having abusive consumption in those consuming alcohol (Table 4). Sex is an important risk factor with increased risk for the men compared to women (AOR = 2.53 [1.38 – 4.68]) as well as the occupational status with jobless people and housemaker associated with a decreased risk of having abusive consumption (AOR = 0.05 [0.01 – 0.47] and AOR = 0.14 [0.02 – 0.78] respectively).

The risk also varied from the different regions, AORs for the different regions of the countries are presented in Table 5. We observed that the *Sud-Ouest* region has a higher risk of consumption compared to the rest of the country. In the *Sahel*, *Est,* and *Haut-Bassin*, the risks of consumption are significantly lower.

Discussion

We report here the results of the first nationally representative survey on the prevalence and risk factors for alcohol consumption in Burkina Faso.

The overall prevalence of people with abusive alcohol consumption is 2.7% [2.0 – 3.7]. This consumption is lower compared to other African countries [12] and the rest of the world [13]. However, this prevalence might be underestimated because even though Burkina Faso is a secular country, the majority of its inhabitants have a religious faith that prohibits alcohol consumption that could prevent some people from declaring their alcohol intake, this might be particularly true in the *Sahel* region. Also, alcohol consumption is blamed by society and could also lead to an under-declaration (positive social perception bias) [38].

We also found differences in the different regions of the country with the highest levels of consumption found in the *Sud-Ouest* region. Altought the results a regional level must be interpreted carefully due to the relative small number of participants included, they are of

For peer review only - http://bmjopen.bmj.com/site/about/guidelines.xhtml

importance for public health. In this region, almost every household produces local beer ('*dolo*') this might explain this observation. The income disparities and the alcohol availability between the different regions could also explain those differences [39]. From a public health perspective, this region also has the highest prevalence of Hepatitis B and C [40]. The inhabitants of these regions seem to cumulate the hepatotoxic risk of Hepatitis and alcohol consumption. We found a null prevalence in the Sahel region, this is an entirely Muslim region and the desirability bias may be more important compared to other regions.

We also found gender differences with an increased risk in men compared to women for low and abusive alcohol consumption but an increased risk of moderate consumption for women. It is important to specify here that, following the WHO guidelines, the thresholds used to define the different levels of alcohol consumption are different for men and women (33 to 50% lower for women compared to men). The risk of abusive consumption in drinkers is more important in men compared to women.

In most countries, the prevalence is higher in men compared to women [19,41], but when adjusting for multiple factors such as social supports and financial aspects, this effect seems less important. Compared to men, more women are lifetime abstainers, drink less, and are less likely to engage in problem drinking. Of note is that women drinking excessively develop more severe medical problems than men. Biological (sex-related) factors, including differences in alcohol pharmacokinetics as well as its effect on brain function and the levels of sex hormones, may contribute to some of those differences [42]. Since pregnant women are particularly vulnerable, we performed a sub-group analysis. A subgroup of 299 out of the 2,449 (12.2%) women was pregnant during the survey, amongst them, 6 (3.9%) reported

For peer review only - http://bmjopen.bmj.com/site/about/guidelines.xhtml

alcohol consumption, which is statistically lower compared to non-pregnant women (12.6%, $p < .001$).

Interestingly we observed that age was significantly associated with alcohol consumption with a gradient effect of age on alcohol consumption, but it seems that the level of consumption is not influenced by gender as the AOR are relatively similar for the different categories. The influence of age on alcohol consumption is still unclear and not well documented in the literature, except for binge drinking, where young adults are the most at risk [43]. However, this result should be interpreted carefully, considering our study design. This could be the results of preventive campaigns as reflected by a cohort effect A large Australian study including 7 cross-sectional waves showed indeed that male cohorts born between 1965 and 1974 and female cohorts born between 1955 and 1974 reported higher rates of drinking participation ($p < 0.05$), while the most recent cohorts (born in the 1990s) had lower rates of participation ($p < 0.01$) [44].

Concerning the risk of switching to an abusive consumption among drinkers only two factors have been identified: the risk is increased in males (as for the general consumption) and decreased with the occupational status, probably due to financial constraints.

The association with tobacco consumption is probably one of the most important from a public health view because of the comorbidities and the double burden it presents for the population. In a previous study, we identified people most at risk of tobacco consumption in Burkina Faso: tobacco smoking among men was significantly associated with increased age and alcohol consumption. Analysis of risk factors for other tobacco use stratified by gender shows that age, education, residence, and alcohol consumption were significantly associated with consumption for women, age, and alcohol consumption for men [28].

For peer review only - http://bmjopen.bmj.com/site/about/guidelines.xhtml

As recommended by the WHO, in this paper we presented risk factors associated with abusive alcohol consumption because of the proven negative effects. It is, however, interesting to note that from a medical point of view a limited and reasonable alcohol consumption could have some health-related benefits: for example, the relation between dementia and cognitive disorders is not linear and limited alcohol consumption has a protective role for dementia [45–47] or the protective effect of alcohol on cardiovascular risk, previously known as *'the French paradox'* [48–50]. However, these results must be interpreted with caution, especially since it is known that even in low doses, alcohol consumption transiently increases the risk of cardiovascular accidents [51]. Another important point is that considering the prevention side experiences showed that adopting a too strong position by prohibiting any consumption or behavior will lead to poor results [52,53]. Considering these two aspects low alcohol consumption could be considered as acceptable. Alcohol policies that regulate the physical availability of alcohol are associated with lower alcohol consumption in low- and middle-income countries [54]. Burkina Faso is at an embryonic stage in this area. Indeed, the country just had its first specifications plan on alcoholic beverages production, importation, and selling adopted in 2020 [55]. This plan was jointly approved by the Ministry of Trade and the companies involved in such activities. Some additional measurements have been adopted by the government. These include the prohibition of advertisements for tobacco and alcohol products and, since September 19, 2019, the government also banned the production, importation, and marketing of liqueurs and other spirit drinks in plastic bags and bottles of less than 30 centiliters to limit the availability [56]. This plan needs to be rigorously implemented first and then rapidly incremented to a stronger legislative law to positively impact behavior and health outcomes.

For peer review only - http://bmjopen.bmj.com/site/about/guidelines.xhtml

The main limitation of this study is that alcohol (and tobacco) consumptions were obtained during interviews and is therefore dependent on the faith of the participants. There is, therefore, both a risk of memory bias and social desirability, probably more marked during pregnancy. It can thus be estimated that the numbers and prevalence obtained in this survey underestimate the actual consumption. Another potential limitation is that some well-known risk factors for alcohol consumption were not included in the study because data on these variables have not been collected during the STEPS survey. Part of such variables is socioeconomic status. It is a transversal study; therefore there is a risk of survival bias indicating that older participants with high consumption may die prematurely due to this consumption [57]. The last limitation is that we used data collected in 2013, these data may not represent the actual situation anymore. We have indeed seen that the government has taken measurements to restrict and limit alcohol consumption, therefore the prevalence may be more important than the current situation. However, in this paper, we mainly focus on the risk factor and we do not think that the risk factor has been much modified between 2013 and today. Another STEP survey was planned for 2020 to have more recent numbers but unfortunately, the survey could not take place due to the COVID-19 pandemic. In the future the data of this survey could be used to monitor the level of consumption and determine if these measurements are working and if other measures must be taken.

Despite these limitations, given the study design (cluster sampling design) and the sample size, the results of this study can be extended to the whole of Burkina Faso.

For peer review only - http://bmjopen.bmj.com/site/about/guidelines.xhtml

Conclusion

In this study, we reported on the burden of alcohol consumption and associated risk factors in a nationally representative sample of adults in Burkina Faso. Our data showed an important burden of alcohol consumption in Burkina Faso. Tobacco consumption is an important modifiable risk factor associated with alcohol consumption. Zone-specific interventions are needed given the higher burden in urban centers and some specific regions such as the *Sud-Ouest* region. Health policies in Burkina Faso must henceforth account for the control of alcohol and tobacco consumption since there is a strong relationship between those two important risk factors of NCDs. Alcohol policies that regulate the physical availability of alcohol are associated with lower alcohol consumption and should, therefore, be implemented in Burkina Faso to reduce the burden of alcohol consumption.

List of abbreviation

DHS: Demographic and Health Surveys

NCD: Non-communicable disease

SSA: sub-Saharan Africa

STEPS: WHO STEPwise approach to surveillance

WHO: World Health Organization

For peer review only - http://bmjopen.bmj.com/site/about/guidelines.xhtml

Declarations

Ethics approval and consent to participate Consent for publication

The protocol of the STEPS survey was reviewed and approved by the Ethics Committee for Health Research of the Ministry of Health of Burkina Faso, which gave clearance in accordance with regulations in force (Deliberation No. 2012–12- 092 of 05 December 2012). Written informed consent was systematically sought and obtained from all participants before inclusion in the study. The confidentiality of study participants was fully respected and the analyses performed did not identify any participant. For this study, we obtained authorization from the General Directorate of Health to reanalyze the data, and the confidentiality of study participants was preserved.

Availability of data and materials

The dataset of the STEPS survey that was used in this research is available at the Ministry of Health upon request. Other STEPS surveys can be obtained through the WHO (https://www.who.int/ncds/surveillance/steps/riskfactor/en/). Any request to reanalyze the data can be directed to Dr Brice Bicaba (bicababrico78@gmail.com). No further additional data are available.

Acknowledgments

The authors are thankful to all the people involved in the STEPS survey. Bruno Bonnechère thanks people who help him during the analysis of the data in Burkina Faso.

For peer review only - http://bmjopen.bmj.com/site/about/guidelines.xhtml

Competing interests

The authors declare that they have no competing interests.

Funding

Bonnechère Bruno received a travel grant from ARES (https://www.ares-ac.be/fr/) to conduct the data analysis in Burkina Faso (second analysis of the STEP survey), interpret the data with local epidemiologists and write the manuscript. Cissé Kadari is funded by ARES. The funding body had no role in study design, data collection and analysis, decision to publish, or preparation of the manuscript. Award/Grant number is not applicable

Author contributions

BB and FKS conceived the study. BB performed the analysis. BB, SS and CK proposed an early draft of the paper. FKS, SK, and ST made substantial contributions to the conception and design, analysis and interpretation of the data. KFS, SK and ST contributed significantly to revise the manuscript. All authors read and approved the final manuscript.

For peer review only

For peer review only - http://bmjopen.bmj.com/site/about/guidelines.xhtml

Figures captions

Figure 1: Consort Diagram describing study participants, exclusions and analytic sample size.

Figure 2 : Prevalence of the level of alcohol consumption in the different regions of Burkina Faso. Vertical lines indicate the mean prevalence for the whole country.

For peer review only

For peer review only - http://bmjopen.bmj.com/site/about/guidelines.xhtml

References

1 GBD 2015 DALYs and HALE Collaborators. Global, regional, and national disability-adjusted life-years (DALYs) for 315 diseases and injuries and healthy life expectancy (HALE), 1990-2015: a systematic analysis for the Global Burden of Disease Study 2015. *Lancet Lond Engl* 2016;**388**:1603–58. doi:10.1016/S0140-6736(16)31460-X

2 Lee I-M, Shiroma EJ, Lobelo F, *et al.* Effect of physical inactivity on major non-communicable diseases worldwide: an analysis of burden of disease and life expectancy. *Lancet Lond Engl* 2012;**380**:219–29. doi:10.1016/S0140-6736(12)61031-9

3 NCD Countdown 2030 collaborators. NCD Countdown 2030: worldwide trends in non-communicable disease mortality and progress towards Sustainable Development Goal target 3.4. *Lancet Lond Engl* 2018;**392**:1072–88. doi:10.1016/S0140-6736(18)31992-5

4 World Health Organization. World Health Statistics 2018: Monitoring health for the SDGs. 2018.

5 Jamison DT, Summers LH, Alleyne G, *et al.* Global health 2035: a world converging within a generation. *Lancet Lond Engl* 2013;**382**:1898–955. doi:10.1016/S0140-6736(13)62105-4

6 Kearney PM, Whelton M, Reynolds K, *et al.* Global burden of hypertension: analysis of worldwide data. *Lancet Lond Engl* 2005;**365**:217–23. doi:10.1016/S0140-6736(05)17741-1

7 Whiteford HA, Degenhardt L, Rehm J, *et al.* Global burden of disease attributable to mental and substance use disorders: findings from the Global Burden of Disease Study 2010. *Lancet Lond Engl* 2013;**382**:1575–86. doi:10.1016/S0140-6736(13)61611-6

8 Afshin A, Micha R, Webb M, *et al.* Effectiveness of Dietary Policies to Reduce Noncommunicable Diseases. In: Prabhakaran D, Anand S, Gaziano TA, *et al.*, eds. *Cardiovascular, Respiratory, and Related Disorders*. Washington (DC): : The International Bank for Reconstruction and Development / The World Bank 2017. http://www.ncbi.nlm.nih.gov/books/NBK525147/ (accessed 10 Sep 2019).

9 Juma PA, Mohamed SF, Matanje Mwagomba BL, *et al.* Non-communicable disease prevention policy process in five African countries. *BMC Public Health* 2018;**18**:961. doi:10.1186/s12889-018-5825-7

10 Shield K, Manthey J, Rylett M, *et al.* National, regional, and global burdens of disease from 2000 to 2016 attributable to alcohol use: a comparative risk assessment study. *Lancet Public Health* 2020;**5**:e51–61. doi:10.1016/S2468-2667(19)30231-2

11 Manthey J, Shield KD, Rylett M, *et al.* Global alcohol exposure between 1990 and 2017 and forecasts until 2030: a modelling study. *The Lancet* 2019;**393**:2493–502. doi:10.1016/S0140-6736(18)32744-2

For peer review only - http://bmjopen.bmj.com/site/about/guidelines.xhtml

12 World Health Organization. Global Status Report on Alcohol and Health—2014. 2014.

13 Shield KD, Rylett M, Gmel G, *et al.* Global alcohol exposure estimates by country, territory and region for 2005--a contribution to the Comparative Risk Assessment for the 2010 Global Burden of Disease Study. *Addict Abingdon Engl* 2013;**108**:912–22. doi:10.1111/add.12112

14 Clausen T, Rossow I, Naidoo N, *et al.* Diverse alcohol drinking patterns in 20 African countries. *Addict Abingdon Engl* 2009;**104**:1147–54. doi:10.1111/j.1360-0443.2009.02559.x

15 Probst C, Shuper PA, Rehm J. Coverage of alcohol consumption by national surveys in South Africa. *Addict Abingdon Engl* 2017;**112**:705–10. doi:10.1111/add.13692

16 Ayano G, Yohannis K, Abraha M, *et al.* The epidemiology of alcohol consumption in Ethiopia: a systematic review and meta-analysis. *Subst Abuse Treat Prev Policy* 2019;**14**:26. doi:10.1186/s13011-019-0214-5

17 Reuters. SABMiller Eyes Africa $2.5bn Investment. 2012.http://www.iol.co.za/business/international/sabmiller-eyes-africa-25bn-investment-1277086 (accessed 8 Jul 2019).

18 African Business. Alcohol Consumption Rising in Africa Despite Obstacles. https://africanbusinessmagazine.com/sectors/retail/alcohol-consumption-rising-in-africa-despite-obstacles/ (accessed 8 Jul 2019).

19 Chaiyasong S, Huckle T, Mackintosh A-M, *et al.* Drinking patterns vary by gender, age and country-level income: Cross-country analysis of the International Alcohol Control Study. *Drug Alcohol Rev* 2018;**37 Suppl 2**:S53–62. doi:10.1111/dar.12820

20 Huckle T, Romeo JS, Wall M, *et al.* Socio-economic disadvantage is associated with heavier drinking in high but not middle-income countries participating in the International Alcohol Control Study. *Drug Alcohol Rev* 2018;**37 Suppl 2**:S63–71. doi:10.1111/dar.12810

21 Martinez P, Røislien J, Naidoo N, *et al.* Alcohol abstinence and drinking among African women: data from the World Health Surveys. *BMC Public Health* 2011;**11**:160. doi:10.1186/1471-2458-11-160

22 Popova S, Lange S, Probst C, *et al.* Actual and predicted prevalence of alcohol consumption during pregnancy in the WHO African Region. *Trop Med Int Health TM IH* 2016;**21**:1209–39. doi:10.1111/tmi.12755

23 Sanou AS, Diallo AH, Holding P, *et al.* Maternal alcohol consumption during pregnancy and child's cognitive performance at 6-8 years of age in rural Burkina Faso: an observational study. *PeerJ* 2017;**5**:e3507. doi:10.7717/peerj.3507

24 Addila AE, Bisetegn TA, Gete YK, *et al.* Alcohol consumption and its associated factors among pregnant women in Sub-Saharan Africa: a systematic review and meta-analysis' as

For peer review only

given in the submission system. *Subst Abuse Treat Prev Policy* 2020;**15**:29. doi:10.1186/s13011-020-00269-3

25 Addo J, Cook S, Galbete C, *et al.* Differences in alcohol consumption and drinking patterns in Ghanaians in Europe and Africa: The RODAM Study. *PloS One* 2018;**13**:e0206286. doi:10.1371/journal.pone.0206286

26 Mosdøl A, Lidal IB, Straumann GH, *et al.* Targeted mass media interventions promoting healthy behaviours to reduce risk of non-communicable diseases in adult, ethnic minorities. *Cochrane Database Syst Rev* 2017;**2**:CD011683. doi:10.1002/14651858.CD011683.pub2

27 Soubeiga JK, Millogo T, Bicaba BW, *et al.* Prevalence and factors associated with hypertension in Burkina Faso: a countrywide cross-sectional study. *BMC Public Health* 2017;**17**:64. doi:10.1186/s12889-016-3926-8

28 Bonnechère B, Cissé K, Millogo Tiéba, *et al.* Tobacco use and associated risk factors in Burkina Faso: Results from a nationwide population-based survey. *BMC Public Health* 2019.

29 Ministère de l'économie et des finances. Projection démographique de 2007 à 2020 par région et province. Institut national de la statistique et de la démographie, Burkina Faso. http://www.cns.bf/IMG/pdf/projections_demographiques_sous_nationales.pdf

30 Institut National de la Statistique et de la Démographie (INSD); ICF International. Enquête Démographique et de Santé et à Indicateurs Multiples du Burkina Faso 2010. 2012.

31 Institut national de la statistique et de la démographie. BURKINA FASO MINISTERE DE L'ECONOMIE ET DES FINANCES: PROJECTIONS DEMOGRAPHIQUES DE 2007 A 2020 PAR REGION ET PROVINCE. http://www.cns.bf/IMG/pdf/projections_demographiques_sous_nationales.pdf

32 Ministère de la santé du Burkina Faso. Rapport de l'enquête nationale sur la prévalence des principaux facteurs de risque communs aux maladies non transmissibles (STEPS 2013). 2014.

33 La Croix A. Données géographiques et identité religieuse au Burkina Faso. Données Géographiques Identité Relig. Au Burkina Faso. https://africa.la-croix.com/statistiques/burkina-faso/ (accessed 28 Feb 2021).

34 Bonnet E, Lechat L, Ridde V. What interventions are required to reduce road traffic injuries in Africa? A scoping review of the literature. *PloS One* 2018;**13**:e0208195. doi:10.1371/journal.pone.0208195

35 WHO. STEPwise approach to chronic disease risk factor surveillance. https://www.who.int/ncds/surveillance/steps/burkina_faso/en/

For peer review only - http://bmjopen.bmj.com/site/about/guidelines.xhtml

36 Millogo T, Bicaba BW, Soubeiga JK, *et al.* Diabetes and abnormal glucose regulation in the adult population of Burkina Faso: prevalence and predictors. *BMC Public Health* 2018;**18**. doi:10.1186/s12889-018-5257-4

37 Kendagor A, Gathecha G, Ntakuka MW, *et al.* Prevalence and determinants of heavy episodic drinking among adults in Kenya: analysis of the STEPwise survey, 2015. *BMC Public Health* 2018;**18**:1216. doi:10.1186/s12889-018-6057-6

38 Jemmott JB, Ditto PH, Croyle RT. Judging health status: effects of perceived prevalence and personal relevance. *J Pers Soc Psychol* 1986;**50**:899–905. doi:10.1037//0022-3514.50.5.899

39 Dixon MA, Chartier KG. Alcohol Use Patterns Among Urban and Rural Residents: Demographic and Social Influences. *Alcohol Res Curr Rev* 2016;**38**:69–77.

40 Meda N, Tuaillon E, Kania D, *et al.* Hepatitis B and C virus seroprevalence, Burkina Faso: a cross-sectional study. *Bull World Health Organ* 2018;**96**:750–9. doi:10.2471/BLT.18.208603

41 Takahashi R, Wilunda C, Magutah K, *et al.* Correlates of alcohol consumption in rural western Kenya: A cross-sectional study. *BMC Psychiatry* 2017;**17**:175. doi:10.1186/s12888-017-1344-9

42 Erol A, Karpyak VM. Sex and gender-related differences in alcohol use and its consequences: Contemporary knowledge and future research considerations. *Drug Alcohol Depend* 2015;**156**:1–13. doi:10.1016/j.drugalcdep.2015.08.023

43 Kuntsche E, Kuntsche S, Thrul J, *et al.* Binge drinking: Health impact, prevalence, correlates and interventions. *Psychol Health* 2017;**32**:976–1017. doi:10.1080/08870446.2017.1325889

44 Livingston M, Raninen J, Slade T, *et al.* Understanding trends in Australian alcohol consumption-an age-period-cohort model. *Addict Abingdon Engl* 2016;**111**:1590–8. doi:10.1111/add.13396

45 Heffernan M, Mather KA, Xu J, *et al.* Alcohol Consumption and Incident Dementia: Evidence from the Sydney Memory and Ageing Study. *J Alzheimers Dis JAD* 2016;**52**:529–38. doi:10.3233/JAD-150537

46 Xu W, Wang H, Wan Y, *et al.* Alcohol consumption and dementia risk: a dose-response meta-analysis of prospective studies. *Eur J Epidemiol* 2017;**32**:31–42. doi:10.1007/s10654-017-0225-3

47 Gutwinski S, Schreiter S, Priller J, *et al.* Drink and Think: Impact of Alcohol on Cognitive Functions and Dementia - Evidence of Dose-Related Effects. *Pharmacopsychiatry* 2018;**51**:136–43. doi:10.1055/s-0043-118664

For peer review only

For peer review only - http://bmjopen.bmj.com/site/about/guidelines.xhtml

48 O'Keefe JH, Bhatti SK, Bajwa A, *et al.* Alcohol and cardiovascular health: the dose makes the poison…or the remedy. *Mayo Clin Proc* 2014;**89**:382–93. doi:10.1016/j.mayocp.2013.11.005

49 Fernández-Solà J. Cardiovascular risks and benefits of moderate and heavy alcohol consumption. *Nat Rev Cardiol* 2015;**12**:576–87. doi:10.1038/nrcardio.2015.91

50 Rehm J, Roerecke M. Cardiovascular effects of alcohol consumption. *Trends Cardiovasc Med* 2017;**27**:534–8. doi:10.1016/j.tcm.2017.06.002

51 Mostofsky E, Chahal HS, Mukamal KJ, *et al.* Alcohol and Immediate Risk of Cardiovascular Events: A Systematic Review and Dose-Response Meta-Analysis. *Circulation* 2016;**133**:979–87. doi:10.1161/CIRCULATIONAHA.115.019743

52 Cyril S, Smith BJ, Renzaho AMN. Systematic review of empowerment measures in health promotion. *Health Promot Int* 2016;**31**:809–26. doi:10.1093/heapro/dav059

53 Gelius P, Rütten A. Conceptualizing structural change in health promotion: why we still need to know more about theory. *Health Promot Int* 2018;**33**:657–64. doi:10.1093/heapro/dax006

54 Cook WK, Bond J, Greenfield TK. Are alcohol policies associated with alcohol consumption in low- and middle-income countries? *Addict Abingdon Engl* 2014;**109**:1081–90. doi:10.1111/add.12571

55 Ministère de l'Industrie, du Commerce et de l'Artisanat. Communiqué MICA. https://www.commerce.gov.bf/accueil (accessed 3 Mar 2021).

56 Burkina24. Production et commercialisation d'alcool au Burkina : Bientôt un cahier de charges. https://www.burkina24.com/2020/07/02/production-et-commercialisation-dalcool-au-burkina-bientot-un-cahier-de-charges/

57 Roerecke M. On bias in alcohol epidemiology and the search for the perfect study. *Addict Abingdon Engl* 2017;**112**:217–8. doi:10.1111/add.13549

For peer review only - http://bmjopen.bmj.com/site/about/guidelines.xhtml

Tables

Table 1: Definition of recoded exposure variables.

Variables	Categories
Age groups	"25 to 34 years old", "35 to 44 years old", "45 to 54 years old", "55 to 64 years old"
Education	"None", "Primary", "Secondary", "Tertiary"
Marital status	"Single", "Married", "Divorced/widowed"
Occupational status	"Wage earner", "Self-employed", "Jobless", "Housemaker"
Alcohol consumption (WHO recommendations) [37]	None: Never intake of alcohol<br>Low: intake of an average quantity of pure alcohol of less than 40 g per day for men and less than 20 g for women<br>Mid: corresponds to taking an average quantity of pure alcohol of between 40 g and 59.9 g per day for men and between 20 g and 39.9 g for women<br>Abusive: intake of an average quantity of pure alcohol greater than or equal to 60 g per day for men and greater than or equal to 40 g for women.

Note than one glass of alcohol (beer, wine, 'dolo') contains 10g of ethanol, the recall period is 30 days.

For peer review only

Table 2: Socio-demographic characteristics of the study sample and prevalence of alcohol and abusive alcohol consumption

Variables	Participants, n (%)	Alcohol consumption [95% CI]				
		None	Low	Mid	Abusive	p-value
**Age groups, years (n= 4,692)**						
25 to 34 years	2,124 (42.0)	80.4 [77.1 – 83.4]	10.6 [8.4 – 13.1]	7.0 [5.5 – 8.8]	1.9 [1.2 – 2.9]	<0.001
35 to 44 years	1,181 (27.9)	74.8 [70.7 – 78.6]	13.1 [10.4 – 16.3]	8.9 [6.8 – 11.5]	3.1 [1.9 – 4.9]	
45 to 54 years	841 (18.6)	71.9 [66.7 – 76.6]	14.8 [11.0 – 19.6]	9.5 [7.1 – 12.5]	3.8 [2.3 – 6.1]	
55 to 64 years	546 (11.5)	67.3 [60.4 – 73.5]	18.3 [14.1 – 23.4]	11.3 [8.3 – 15.3]	3.0 [1.5 – 6.2]	
**Sex (n = 4,692)**						
Female	2,436 (54.3)	79.6 [75.9 – 82.8]	8.6 [6.3 – 11.7]	10.8 [8.8 – 13.3]	0.9 [0.6 – 1.5]	<0.001
Male	2,256 (45.7)	71.2 [67.3 – 74.9]	18.1 [15.5 – 21.1]	5.8 [4.6 – 7.1]	4.8 [3.4 – 6.8]	
**Living environment (n = 4,692)**						
Urban	945 (24.9)	72.9 [67.0 – 78.1]	12.7 [8.4 – 18.6]	10.0 [7.3 – 13.5]	4.3 [2.5 – 7.2]	0.001
Rural	3,747 (75.1)	76.7 [72.9 – 80.1]	13.1 [10.8 – 15.6]	8.0 [6.4 – 9.9]	2.2 [1.5 – 3.1]	
**Education (n = 4,684)**						
None	3,622 (77.3)	77.9 [74.2 – 81.2]	12.2 [9.9 – 14.8]	7.8 [6.2 – 9.7]	2.0 [1.4 – 2.9]	<0.001
Primary	728 (15.3)	71.9 [66.8 – 76.6]	15.6 [11.9 – 20.1]	8.8 [6.7 – 11.5]	3.6 [2.0 – 6.3]	

For peer review only - http://bmjopen.bmj.com/site/about/guidelines.xhtml

Secondary	334 (7.3)	60.7 [52.7 – 68.1]	15.7 [11.7 – 20.7]	15.3 [11.2 – 20.4]	8.2 [4.5 – 14.6]	
**Marital status (n = 4,688)**						
Single	333 (6.8)	68.6 [59.5 – 76.4]	16.9 [11.8 – 23.6]	9.2 [6.1 – 13.8]	5.2 [2.6 – 10.1]	<0.001
Married	4,043 (87.2)	76.5 [73.1 – 79.5]	12.8 [10.6 – 15.4]	8.2 [6.7 – 9.9]	2.4 [1.8 – 3.3]	
Divorced/Widowed	311 (5.9)	73.2 [66.2 – 79.2]	10.4 [6.9 – 9.9]	12.5 [8.8 – 17.6]	3.8 [1.6 – 8.5]	
**Occupational status (n = 4,692)**						
Wage earner	281 (6.3)	62.2 [55.3 – 68.6]	15.1 [10.7 – 20.9]	12.7 [8.5 – 18.5]	9.9 [5.8 – 16.2]	<0.001
Self-employed	3,249 (66.4)	75.9 [72.2 – 79.3]	13.2 [11.2 – 15.5]	7.9 [6.3 – 9.8]	2.9 [2.1 – 4.3]	
Jobless	125 (2.9)	77.1 [67.5 – 84.6]	11.8 [6.7 – 20.0]	10.4 [5.7 – 18.2]	0.5 [0.1 – 3.4]	
Housemaker	1,037 (24.5)	78.7 [72.8 – 83.6]	11.9 [7.6 – 18.2]	8.9 [6.8 – 11.6]	0.4 [0.1 – 1.4]	
**Tobacco consumption (n = 4,692)**						
No	4,217 (90.8)	77.3 [73.9 – 80.3]	12.1 [10.0 – 14.6]	8.4 [6.9 – 10.1]	2.1 [1.6 – 2.9]	<0.001
< 5 cigs/day	210 (3.8)	67.7 [59.5 – 74.9]	23.9 [17.6 – 31.5]	1.9 [0.8 – 4.5]	6.4 [3.2 – 12.6]	
5 – 10 cigs/day	184 (3.6)	59.7 [50.8 – 67.9]	19.8 [13.5 – 28.1]	13.8 [8.5 – 21.4]	6.6 [3.2 – 13.0]	
> 10 cigs/day	81 (1.7)	48.1 [32.8 – 63.7]	18.4 [10.7 – 29.8]	17.9 [9.8 – 30.1]	15.5 [4.6 – 40.7]	

p-values indicate the results of the chi-square tests.

For peer review only - http://bmjopen.bmj.com/site/about/guidelines.xhtml

Table 3: Factors associated with alcohol and abusive alcohol consumptions in population aged 25 – 64 years.

Variables	Low consumption		Mid consumption		Abusive consumption	
	n	AOR [95% CI]	n	AOR [95% CI]	n	AOR [95% CI]
**Age groups**						
25 years to 34 years (n = 2,224)	221	Ref	151	Ref	41	Ref
35 years to 44 years (n = 1,181)	161	**1.46 [1.09 – 1.95]***	112	**1.55 [1.14 – 2.12]****	30	**1.86 [1.06 – 3.27]***
44 years to 54 years (n = 841)	123	**1.79 [1.34 – 2.40]*****	81	**1.81 [1.25 – 2.62]****	30	**2.67 [1.36 – 5.25]****
55 to 64 (n =546)	105	**2.36 [1.59 – 3.51]*****	55	**2.50 [1.54 – 4.07]*****	19	**2.37 [1.01 – 5.92]***
**Sex**						
Female (n = 2,436)	204	Ref	270	Ref	22	Ref
Male (n = 2,256)	406	**2.68 [1.96 – 3.67]*****	129	**0.38 [0.26 – 0.55]*****	98	**2.89 [1.56 – 2.37]****
**Living environment**						
Urban (n = 945)	111	Ref	109	Ref	30	Ref
Rural (n = 3,747)	499	1.18 [0.59 – 2.33]	290	1.12 [0.68 – 1.87]	90	0.76 [0.40 – 1.45]
**Education**						
None (n = 3,622)	452	Ref	281	Ref	81	Ref
Primary (n = 728)	112	**1.47 [1.05 – 2.06]***	67	1.47 [0.99 – 2.18]	19	1.60 [0.81 – 3.18]
Secondary (n = 334)	46	**2.08 [1.07 – 4.03]***	49	**2.97 [1.48 – 5.92]****	19	**3.50 [1.34 – 9.15]****

For peer review only - http://bmjopen.bmj.com/site/about/guidelines.xhtml

**Marital status**						
Single (n = 333)	57	Ref	31	Ref	17	Ref
Married (n = 4,043)	514	0.66 [0.40 – 1.10]	93	0.74 [0.43 – 1.30]	93	0.56 [0.23 – 1.36]
Divorced/Widowed (n = 311)	39	0.56 [0.29 – 1.08]	10	0.83 [0.42 – 1.63]	10	1.01 [0.31 – 3.33]
**Occupational status**						
Wage earner (n = 281)	47	Ref	35	Ref	20	Ref
Self-employed (n = 3,249)	436	1.10 [0.58 – 2.06]	246	0.90 [0.53 – 1.51]	95	0.65 [0.26 – 1.67]
Jobless (n = 125)	16	0.75 [0.37 – 1.53]	15	0.77 [0.33 – 1.76]	1	**0.05 [0.01 – 0.44]** **
House maker (n = 1,037)	111	2.20 [0.95 – 5.08]	103	0.74 [0.41 – 1.33]	4	0.23 [0.04 – 1.15]
**Tobacco consumption**						
No (n = 4,217)	512	Ref	354	Ref	86	Ref
< 5 cigs/day (n = 210)	47	**1.72 [1.13 – 2.62]***	6	0.52 [0.21 – 1.31]	14	**2.42 [1.07 – 5.63]***
5 – 10 cigs/day (n = 184)	36	1.60 [0.98 – 2.62]	23	**4.20 [2.25 – 7.83]*****	11	**2.48 [1.06 – 5.79]***
> 10 cigs/day (n = 81)	15	1.77 [0.83 – 3.75]	16	**6.08 [2.75 – 13.4]*****	9	**6.58 [1.96 – 22.11]****

* p = 0.05, ** p = 0.01, *** p < 0.001

For peer review only - http://bmjopen.bmj.com/site/about/guidelines.xhtml

Table 4: Factors associated with abusive alcohol consumptions in population aged 25 – 64 years consuming alcohol.

Variables	Alcohol consumption	Abusive alcohol consumption	
	N	N	AOR [95%CI]
**Age groups**			
25 years to 34 years	413	41	Ref
35 years to 44 years	303	30	1.55 (0.84 – 2.86)
44 years to 54 years	234	30	1.91 (0.95 – 3.85)
55 to 64	179	19	0.99 (0.38 – 2.57)
**Sex**			
Female	496	22	Ref
Male	633	98	**2.53 (1.38 – 4.68)****
**Living environment**			
Urban	250	30	Ref
Rural	879	90	0.68 (0.34 – 1.36)
**Education**			
None	814	81	Ref
Primary	198	19	0.89 (0.43 – 1.86)
Secondary	114	19	1.25 (0.48 – 3.25)
**Marital status**			
Single	105	17	Ref
Married	930	93	0.70 (0.32 – 1.53)
Divorced/Widowed	94	10	1.41 (0.45 – 4.46)
**Occupational status**			
Wage earner	102	20	Ref
Self-employed	777	95	0.51 (0.19 – 1.50)
Jobless	32	1	**0.05 (0.01 – 0.47)****
House maker	218	4	**0.14 (0.02 – 0.78)***
**Tobacco consumption**			
No	952	86	Ref
< 5 cigs/day	67	14	1.71 (0.68 – 4.26)
5 – 10 cigs/day	70	11	1.20 (0.51 – 2.86)

For peer review only - http://bmjopen.bmj.com/site/about/guidelines.xhtml

For peer review only

> 10 cigs/day	40	9	2.26 (0.63 – 8.05)

* p = 0.05, ** p = 0.01, *** p < 0.001

For peer review only

For peer review only - http://bmjopen.bmj.com/site/about/guidelines.xhtml

Table 5: Risk of alcohol consumption in the different regions of the country.

Region	N	Alcohol consumption	
		n	AOR (95%CI)
Centre	548	168	Ref
Boucle du Mouhoun	469	147	1.23 [0.63 – 2.39]
Cascades	166	45	0.58 [0.28 – 1.19]
Centre-Est	398	72	0.51 [0.24 – 1.06]
Centre-Nord	434	87	0.67 [0.35 – 1.29]
Centre-Ouest	390	121	0.94 [0.45 – 1.99]
Centre-Sud	217	51	0.66 [0.28 – 1.54]
Est	376	63	0.40 [0.22 – 0.74]**
Haut-Bassin	509	89	0.39 [0.24 – 0.66]***
Nord	420	72	0.66 [0.28 – 1.58]
Plateau Central	236	62	1.01 [0.37 – 2.75]
Sahel	315	4	0.02 [0.01 – 0.15]***
Sud-Ouest	214	148	5.63 [1.98 – 16.01]***

Model is adjusted for age, sex, living environment, education, marital status, occupational status and tobacco consumption. Alcohol consumption includes the different levels of consumption

* p = 0.05, ** p = 0.01, *** p < 0.001

For peer review only

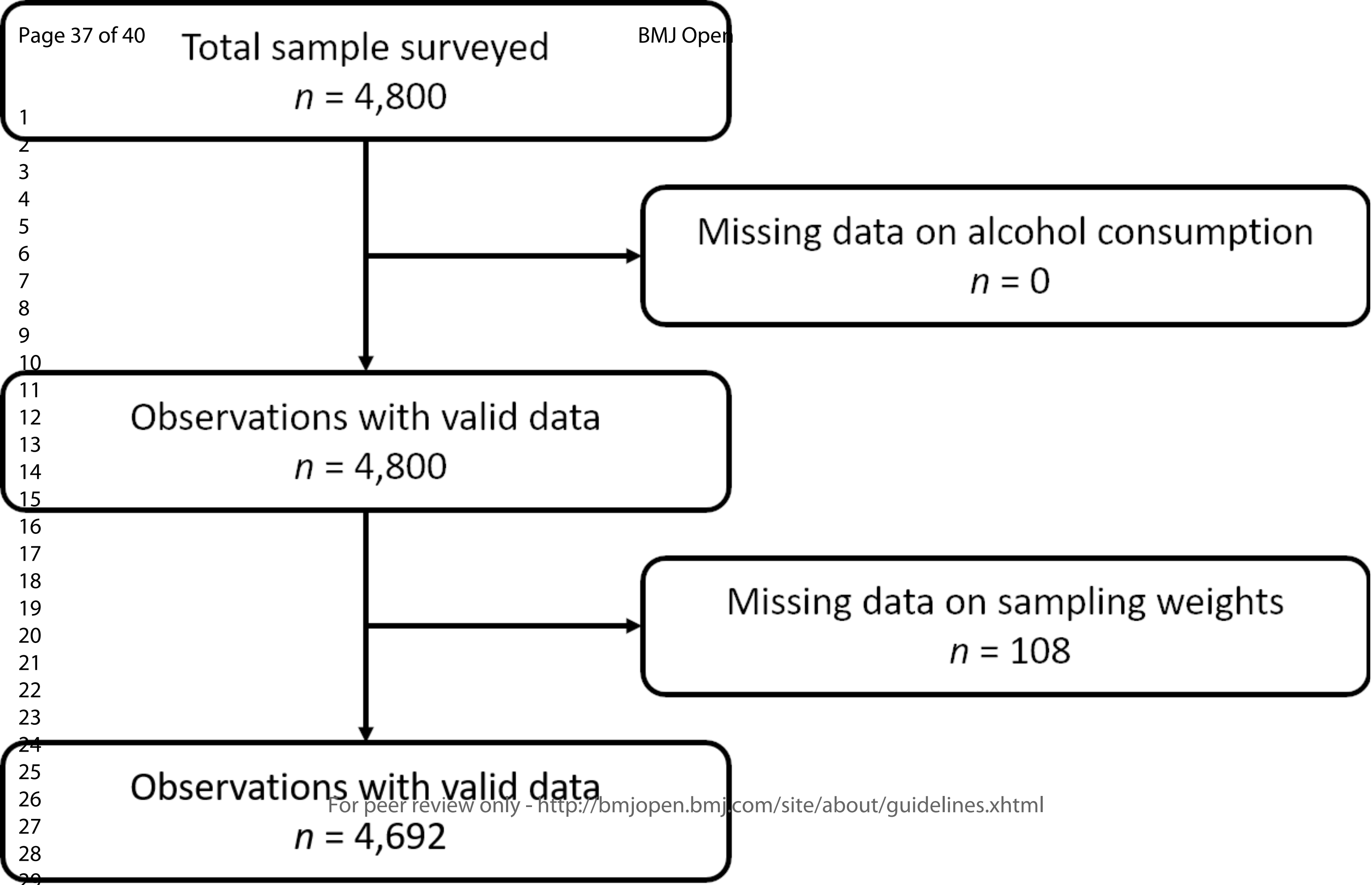

For peer review only - http://bmjopen.bmj.com/site/about/guidelines.xhtml

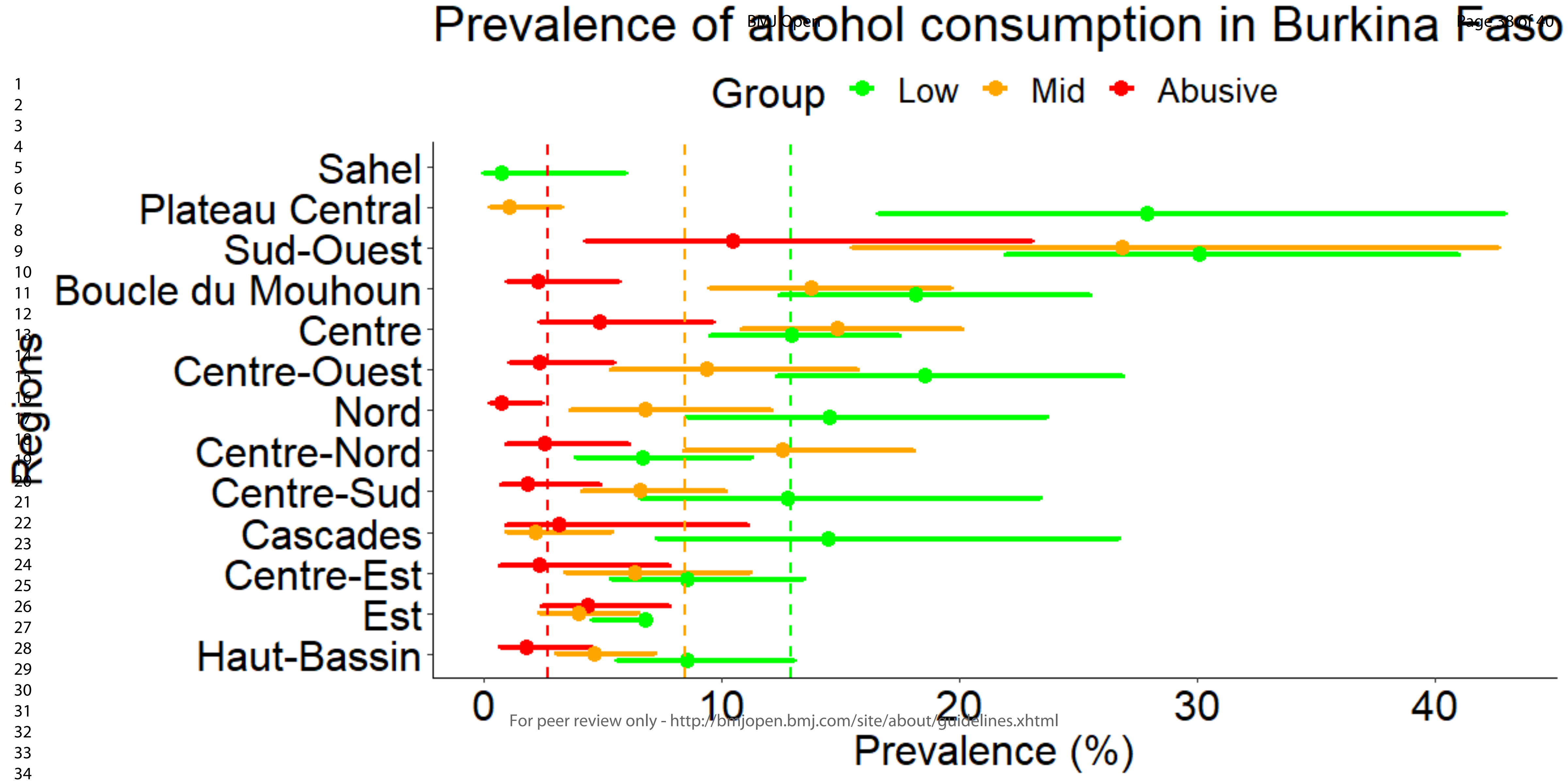
Prevalence of alcohol consumption in Burkina Faso
Group
Low
Mid
Abusive
Regions
Sahel
Plateau Central
Sud-Ouest
Boucle du Mouhoun
Centre
Centre-Ouest
Nord
Centre-Nord
Centre-Sud
Cascades
Centre-Est
Est
Haut-Bassin
0
10
20
30
40
Prevalence (%)

STROBE Statement—Checklist of items that should be included in reports of ***cross-sectional studies***

	Item No	Recommendation	Page
**Title and abstract**	1	(*a*) Indicate the study's design with a commonly used term in the title or the abstract	Yes
		(*b*) Provide in the abstract an informative and balanced summary of what was done and what was found	Yes
**Introduction**			
Background/rationale	2	Explain the scientific background and rationale for the investigation being reported	p.3
Objectives	3	State specific objectives, including any prespecified hypotheses	p.4
**Methods**			
Study design	4	Present key elements of study design early in the paper	Data source on p.5-6, sampling p.6
Setting	5	Describe the setting, locations, and relevant dates, including periods of recruitment, exposure, follow-up, and data collection	p.6
Participants	6	(*a*) Give the eligibility criteria, and the sources and methods of selection of participants	STEPs survey defined by WHO (p.6)
Variables	7	Clearly define all outcomes, exposures, predictors, potential confounders, and effect modifiers. Give diagnostic criteria, if applicable	p.7 and Table 1 and 2
Data sources/ measurement	8*	For each variable of interest, give sources of data and details of methods of assessment (measurement). Describe comparability of assessment methods if there is more than one group	NA
Bias	9	Describe any efforts to address potential sources of bias	NA
Study size	10	Explain how the study size was arrived at	p.5-6
Quantitative variables	11	Explain how quantitative variables were handled in the analyses. If applicable, describe which groupings were chosen and why	Definition of recoded exposure variables is presented in Table 1
Statistical methods	12	(*a*) Describe all statistical methods, including those used to control for confounding	p.7-8
		(*b*) Describe any methods used to examine subgroups and interactions	Multivariable logistic regression models and adjusted OR have been used (p.8 and in the results section)
		(*c*) Explain how missing data were addressed	Flow of the study participants is presented in Figure 1
		(*d*) If applicable, describe analytical methods taking account of sampling strategy	All data were weighted according to the cluster sampling design of the survey using strata and primary

For peer review only - http://bmjopen.bmj.com/site/about/guidelines.xhtml

			sampling units to allow the sample to be nationally representative (p.6)
		(*e*) Describe any sensitivity analyses	NA
**Results**			
Participants	13*	(a) Report numbers of individuals at each stage of study—eg numbers potentially eligible, examined for eligibility, confirmed eligible, included in the study, completing follow-up, and analysed	Figure 1
		(b) Give reasons for non-participation at each stage	NA
		(c) Consider use of a flow diagram	Figure 1
Descriptive data	14*	(a) Give characteristics of study participants (eg demographic, clinical, social) and information on exposures and potential confounders	Table 2
		(b) Indicate number of participants with missing data for each variable of interest	Figure 1
Outcome data	15*	Report numbers of outcome events or summary measures	Table 3
Main results	16	(*a*) Give unadjusted estimates and, if applicable, confounder-adjusted estimates and their precision (eg, 95% confidence interval). Make clear which confounders were adjusted for and why they were included	Table 3
		(*b*) Report category boundaries when continuous variables were categorized	Table 1
		(*c*) If relevant, consider translating estimates of relative risk into absolute risk for a meaningful time period	NA
Other analyses	17	Report other analyses done—eg analyses of subgroups and interactions, and sensitivity analyses	Multivaiate analysis are presented in Table 3 and explained in the method p.6-8
**Discussion**			
Key results	18	Summarise key results with reference to study objectives	First paragraphs of the discussion (p.11)
Limitations	19	Discuss limitations of the study, taking into account sources of potential bias or imprecision. Discuss both direction and magnitude of any potential bias	Start of the discussion (p.11)
Interpretation	20	Give a cautious overall interpretation of results considering objectives, limitations, multiplicity of analyses, results from similar studies, and other relevant evidence	p.11-12
Generalisability	21	Discuss the generalisability (external validity) of the study results	p.12-13
**Other information**			

For peer review only

Funding	22	Give the source of funding and the role of the funders for the present study and, if applicable, for the original study on which the present article is based	None

*Give information separately for exposed and unexposed groups.

Note: An Explanation and Elaboration article discusses each checklist item and gives methodological background and published examples of transparent reporting. The STROBE checklist is best used in conjunction with this article (freely available on the Web sites of PLoS Medicine at http://www.plosmedicine.org/, Annals of Internal Medicine at http://www.annals.org/, and Epidemiology at http://www.epidem.com/). Information on the STROBE Initiative is available at www.strobe-statement.org.

For peer review only

For peer review only - http://bmjopen.bmj.com/site/about/guidelines.xhtml
